# Evolution of diverse host infection mechanisms delineates an adaptive radiation of lampsiline freshwater mussels centered on their larval ecology

**DOI:** 10.7717/peerj.12287

**Published:** 2021-11-16

**Authors:** Trevor L. Hewitt, Amanda E. Haponski, Diarmaid Ó. Foighil

**Affiliations:** Department of Ecology and Evolutionary Biology and Museum of Zoology, University of Michigan - Ann Arbor, Ann Arbor, MI, United States of America

**Keywords:** Phylogenomics, Unionidae, RADseq, Parasitism

## Abstract

North American watersheds contain a high diversity of freshwater mussels (Unionoida). During the long-lived, benthic phase of their life cycle, up to 40 species can co-occur in a single riffle and there is typically little evidence for major differences in their feeding ecology or microhabitat partitioning. In contrast, their brief parasitic larval phase involves the infection of a wide diversity of fish hosts and female mussels have evolved a spectrum of adaptations for infecting host fish with their offspring. Many species use a passive broadcast strategy: placing high numbers of larvae in the water column and relying on chance encounters with potential hosts. Many other species, including most members of the Lampsilini, have a proactive strategy that entails the use of prey-mimetic lures to change the behavior of the hosts, *i.e.*, eliciting a feeding response through which they become infected. Two main lure types are collectively produced: mantle tissue lures (on the female’s body) and brood lures, containing infective larvae, that are released into the external environment. In this study, we used a phylogenomic approach (ddRAD-seq) to place the diversity of infection strategies used by 54 North American lampsiline mussels into an evolutionary context. Ancestral state reconstruction recovered evidence for the early evolution of mantle lures in this clade, with brood lures and broadcast infection strategies both being independently derived twice. The most common infection strategy, occurring in our largest ingroup clade, is a mixed one in which mimetic mantle lures are apparently the predominant infection mechanism, but gravid females also release simple, non-mimetic brood lures at the end of the season. This mixed infection strategy clade shows some evidence of an increase in diversification rate and most members use centrarchids (*Micropterus* & *Lepomis* spp.) as their predominant fish hosts. Broad linkage between infection strategies and predominant fish host genera is also seen in other lampsiline clades: worm-like mantle lures of *Toxolasma* spp. with sunfish (*Lepomis* spp.); insect larvae-like brood lures (*Ptychobranchus* spp.), or mantle lures (*Medionidus* spp., *Obovaria* spp.), or mantle lures combined with host capture (*Epioblasma* spp.) with a spectrum of darter (*Etheostoma* & *Percina* spp.) and sculpin (*Cottus* spp.) hosts, and tethered brood lures (*Hamiota* spp.) with bass (*Micropterus* spp.). Our phylogenetic results confirm that discrete lampsiline mussel clades exhibit considerable specialization in the primary fish host clades their larvae parasitize, and in the host infection strategies they employ to do so. They are also consistent with the hypothesis that larval resource partitioning of fish hosts is an important factor in maintaining species diversity in mussel assemblages. We conclude that, taking their larval ecology and host-infection mechanisms into account, lampsiline mussels may be legitimately viewed as an adaptive radiation.

## Introduction

Adaptive radiation is a form of speciation, enabled by ecological opportunity, in which lineages evolve divergent ecologies and phenotypes to exploit distinct ecological niches (Schluter, 2000; Gavrilets & Losos, 2009). This process is widespread in nature and there are many famous examples of adaptive radiations including Darwin’s finches, cichlid fishes in the East African Great Lakes, and Caribbean anoles (Grant, 1999; Schluter, 2000; Seehausen, 2006). The classic concept of adaptive radiation involves relatively rapid speciation with highly conspicuous phenotypic and ecological differentiation (Schluter, 2000). However, in recent years, these criteria have been expanded to include radiations that have developed over longer temporal scales (Losos, 2010; Arbour & López-Fernández, 2016) as well as radiations characterized by cryptic ecological (Pillon et al., 2014) and phenotypic divergence (Gittenberger & Gittenberger, 2011).

At first glance, most members of the 298 species of unionid mussels found throughout the US and Canada (Williams et al., 2017) would not appear to meet adaptive radiation expectations with regard to ecological distinctiveness. Up to 40 species can co-occur in a single riffle (Haag & Warren, 1998), but there is little evidence for obvious microhabitat partitioning in multispecies aggregations (Strayer, 1981; Strayer & Ralley, 1993), and their nutrition is derived from a combination of ingested sediments (Nichols et al., 2005) and suspended particles (Nichols & Garling, 2000; Vaughn, Nichols & Spooner, 2008). Previous studies have found little evidence of significant resource partitioning in diet among co-occurring species (Coker et al., 1921; Bronmark & Malmqvist, 1982; Raikow & Hamilton, 2001), although a recent study by Tran & Ackerman (2019) found some evidence of differential clearance rates of some planktonic microalgal species in flowing conditions and Atkinson, Ee & Pfeiffer (2020) found variation in tissue stoichiometry among unionid mussels that correlate with phylogeny. The consensus view (Coker et al., 1921; Bronmark & Malmqvist, 1982; Rashleigh & De Angelis, 2007; Vaughn, Nichols & Spooner, 2008; Haag, 2012) is that post-larval resource partitioning alone is an insufficient mechanism to explain the persistence of diverse mussel assemblages in intact US and Canadian rivers.

**Figure 1:**
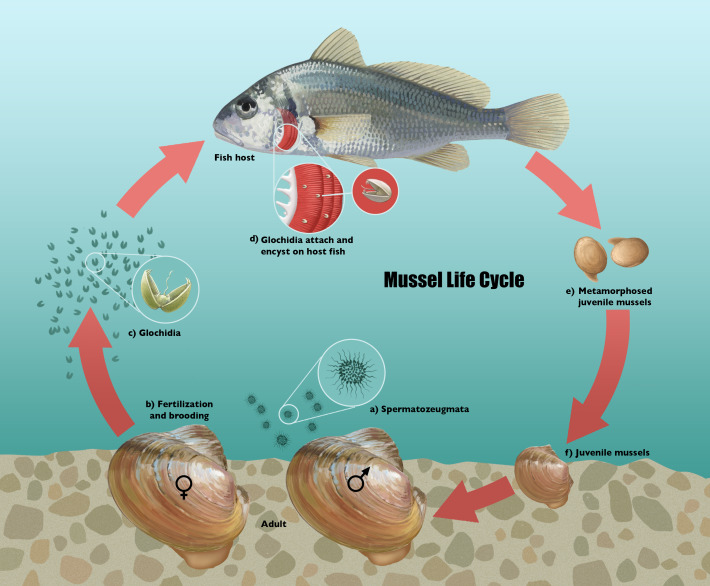
Life cycle diagram of freshwater mussels. Illustration depicting the general life cycle of unionid mussels using *Potamilus ohiensis* as an exemplar. (A) Male mussels release spermatozeugmata into the water column, (B) spermatozeugmata enter female mantel cavity via incurrent siphon to fertilize brooded eggs, (C) parasitic larvae (glochidia) are released into the water column, (D) glochidia attach and encyst on host fish *Aplodinotus grunniens*, (E) metamorphosed juvenile mussels detach from the host, (F) juvenile mussels assume the prolonged benthic phase of the life cycle. Illustration by John Megahan.

The above studies concern the habitat preferences and feeding ecology of the long-lived, macroscopic, post-larval stage of the unionid life cycle (Fig. 1). However, once details of their larval life history and reproductive ecology are taken into account, a large amount of ecological and phenotypic divergence is apparent in this group (Barnhart, Haag & Roston, 2008; Haag, 2012). Uniquely among bivalves, freshwater mussel (Unionoida) larvae are obligate, short-term parasites of fishes (Bogan, 2007; Barnhart, Haag & Roston, 2008; Haag, 2012). This early ontogeny is thought to have evolved as an upstream dispersal mechanism (Watters, 2001; Araujo, Cámara & Ramos, 2002; Barnhart, Haag & Roston, 2008). Co-occurring freshwater mussel species may differ substantially in the fishes used as hosts, the degree of host specialization, the host infection mechanisms used by gravid females, and the seasonality of host infection (Barnhart, Haag & Roston, 2008; Haag, 2012; Cummings & Watters, 2017; Hewitt, Wood & Foighil, 2019). Rashleigh & De Angelis (2007) used ecological modeling to examine partitioning of host use as a mechanism for coexistence in freshwater mussels and found that coexistence via competition for host fish was possible given (1) a high diversity of fish species in the environment; and (2) the ability to target specific fish hosts in the environment. The latter criterion rules out clades largely composed of known fish host generalists such as the subfamilies Unioninae (Barnhart, Haag & Roston, 2008). For fish host specialists, however, we predict that this hypothesized ecological process (Rashleigh & De Angelis, 2007), if valid over longer timescales, would lead to the evolution of adaptive radiations centered on the brief larval life history stage, and characterized by the evolution of host specialization and of specialized host-infection behaviors.

**Figure 2:**
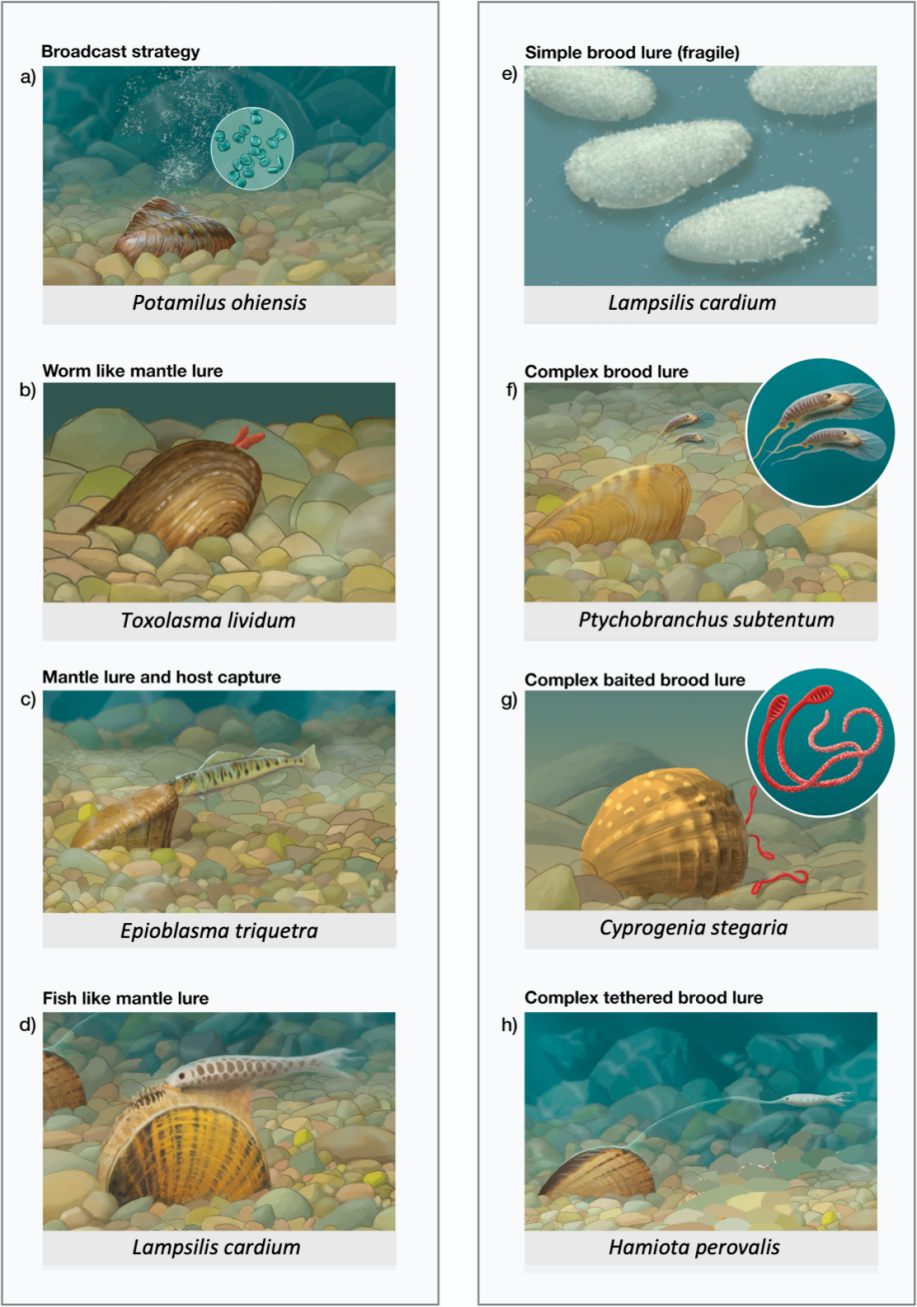
Panel depicting many of the common host infection strategies used by North American freshwater mussels. Illustrations representing most of the primary host infection strategies found in the Lampisilini tribe of North American unionid mussels: (A) broadcast larval release, found in members of the genera *Cyrtonaias, Glebula*, *Leptodea*, *Potamilus* and *Truncilla*, (B) mantle lures in the genus *Toxolasma*–vermiform prey mimic, (C) mantle lure (too small to see here) with associated host capture in the genus *Epioblasma*, (D) mantle lure in the genus *Lampsilis*–piscine prey mimic, (E) simple brood lures, composed of individual marsupia that rapidly break up, released by the genera *Lampsilis, Ligumia, Venustaconcha, Villosa*, *Sagittunio, Cambarunio,* and *Leaunio* (F) complex brood lures in the genus *Ptychobranchus*–larval insect mimic, (G) baited brood lures (white dots are individual larvae) released by the genus *Cyprogenia* (H) tethered complex brood lure in the genus *Hamiota*–piscine prey mimic. Illustrations by John Megahan.

The goal of our study is to test that prediction by analyzing the evolutionary history of host preference and host infection mechanisms in 54 species of lampsiline mussels using the first genomic (ddRAD-seq) phylogeny of the group. We chose this clade because of its high diversity, the availability of extensive background information about host fish specificity (Barnhart, Haag & Roston, 2008; Cummings & Watters, 2017), and, most importantly, because they are predominantly specialist parasites (Haag & Warren, 1998). A given species will typically specialize on a few closely related fish taxa as hosts, *e.g*., darters, or basses, or drum, or sculpins, or percids. They also have a wide diversity of well-documented host fish infection mechanisms. Some species use broadcast release, which relies on passive distribution of larvae in the water column to contact and infect a host (Fig. 2A), but most species have a proactive strategy that entails the use of lures by gravid females to elicit a host feeding response through which they become infected. There are two main lure types (Lefevre & Curtis, 1912; Barnhart, Haag & Roston, 2008): mantle tissue lures on the female’s body (Figs. 2B–2D) and brood lures (*i.e.*, conglutinates and superconglutinates) containing larvae, that are released into the environment (Figs. 2E–2H).

Brood lures are encapsulated aggregates of larvae that form in the female gill demibranch marsupia (Lefevre & Curtis, 1912; Barnhart, Haag & Roston, 2008) and range in complexity from simple, fragile structures that break up upon release (Fig. 2E), to durable aggregations with striking mimicry of prey items including insect larvae (Fig. 2F) and fish fry, to baited worm-like lures partitioned into non-infective and infective sections (Fig. 2G), to tethered lures that resemble prey fish (Barnhart, Haag & Roston, 2008; Haag, 2012). Many lampsiline species employ a mixed strategy that involves mantle lure displays (Fig. 2D) for most of the infection season (usually late spring/early summer) and release of simple non-mimetic brood lures (Fig. 2E) at its end (Corey, Dowling & Strayer, 2006; Barnhart, Haag & Roston, 2008).

An earlier study by Zanatta & Murphy (2006) used a mitochondrial phylogeny to investigate the evolution of host infection strategies in 49 lampsiline species. They recovered evidence for an early evolution of mantle lures in this clade together with a number of secondary losses, in some cases involving the evolution of brood lures (conglutinates/superconglutinates), but many higher-level relationships in their mitochondrial gene trees were poorly supported. We built on their pioneering study by constructing the first genomic lampsiline phylogeny in order to place the diversity of host use, and host infection strategies, into a robust evolutionary context. We were also interested in testing for evidence of a cryptic adaptive radiation, centered on the brief, microscopic, and ecologically diverse, parasitic larval life history stage of this clade, but also incorporating maternal host infection mechanisms.

## Materials & Methods

### Sample collection

Our sampling strategy, for both ingroup and outgroup taxa, was primarily guided by the Zanatta & Murphy (2006) study, although we were not successful in obtaining, and/or genotyping, all of the species they included. Tissues samples from a total of 84 species were collected from the field (*N* = 13) as well as obtained from various research collections (*N* = 71) including the Illinois Natural History Survey, The University of Florida, North Carolina Museum of Natural Sciences, and from the Alabama Aquatic Biodiversity Center. Our final dataset consisted of 109 sequenced individuals representing 54 species across 22 different genera (Table 1).

**Table 1:** Summary table of samples used, life history traits, and summary data for genomic sequencing. Freshwater mussel species included in the phylogenomic analysis, including their host infection strategy, preferred host, total number of illumina reads, total number of clusters, number of consensus reads and total number of loci included in the assembly at an 85% clustering threshold and 25% samples per loci.

Species name	Infection strategy	Host information	Tissue source	Museum ID	Raw reads	Total clusters	Consensus reads	Loci in assembly
*Amblema plicata*	Broadcast	Generalist	Collected by T. Hewitt	306255	2900279	998124	57759	301
*Cambarunio taeniatus*	Mantle lure and Simple brood lure	Bass	NCS	29180	1472633	414265	32713	1004
*Cyrtonaias tampicoensis*	Broadcast	Gar	UF	438173	858098	339345	18540	208
*Epioblasma triquetra*	Mantle lure and Host Trapping	Darter/Sculpin	INHS	36609	5459944	1469677	64027	1678
*Eurynia dilatata*	Broadcast	Generalist	Collected by T. Hewitt	306256	790501	323262	21107	96
*Glebula rotundata*	Broadcast	Sunfish	UF	440636	1070046	557092	25673	303
*Hamiota altilis*	Mantle lure; tethered, complex brood lure	Bass/Sunfish	From Paul Johnson	306257	5387472	1266412	64930	1827
*Hamiota australus*	Tethered, complex brood lure	Bass	UF	441239	3109960	1048442	49094	1494
*Hamiota perovalis*	Tethered, complex brood lure	Bass	From Paul Johnson	306258	5270101	1222099	62362	1826
*Hamiota subangulata*	Tethered, complex brood lure	Bass	UF	438064	668819	207361	20455	722
*Lampsilis bracteata*	Mantle lure and Simple brood lure	Bass	UF	439084	2568126	602005	45170	1594
*Lampsilis cardium*	Mantle lure and Simple brood lure	Bass	Collected by J. Bergner	306259	7216326	2506439	67346	2545
*Lampsilis fasciola*	Mantle lure and Simple brood lure	Bass	Collected by T. Hewitt	306260	3435913	870542	55060	3816
*Lampsilis floridensis*	Mantle lure and Simple brood lure	Bass	UF	340525	3303826	1045716	53781	1595
*Lampsilis higginsi*	Mantle lure and Simple brood lure	Bass	INHS	49425	1009895	330086	13435	512
*Lampsilis hydiana*	Mantle lure and Simple brood lure	Bass	UF	440994	2000552	504555	44904	1743
*Lampsilis ornata*	Mantle lure and Simple brood lure	Bass	UF	438031	4893511	1455910	64521	2177
*Lampsilis ovata*	Mantle lure and Simple brood lure	Bass	UF	438255	1807208	453929	40479	1935
*Lampsilis radiata*	Mantle lure and Simple brood lure	Bass and perch	UF	439013	800488	170092	26694	1262
*Lampsilis satruna*	Mantle lure and Simple brood lure	Bass	UF	441167	4904722	916074	63718	2328
*Lampsilis siliquoidea*	Mantle lure and Simple brood lure	Bass	INHS	25963	2111249	685663	42797	1786
*Lampsilis splendida*	Mantle lure and Simple brood lure	Bass	UF	438354	1149372	286475	28129	1237
*Lampsilis straminea*	Mantle lure and Simple brood lure	Bass	UF	383152	4914716	1562952	66297	2123
*Lampsilis virescens*	Mantle lure and Simple brood lure	Bass	Paul Johnson	306261	4169896	708955	57290	2043
*Leaunio umbrans*	Mantle lure and Simple brood lure	Sunfish/Sculpin	UF	438189	5607023	1832948	69194	1738
*Leaunio vanuxemensis*	Mantle lure and Simple brood lure	Sculpin	UF	438796	1120139	366899	18117	504
*Lemiox rimosus*	Mantle lure	Darter/Sculpin	NCS	47243	1911799	434117	38814	460
*Leptodea fragilis*	Mantle lure	Drum	INHS	79830	3519359	1143382	54580	484
*Leptodea ochracea*	Broadcast	white perch	UF	438459	287978	107862	6669	82
*Ligumia recta*	Mantle lure and Simple brood lure	Walleye	UF	438249	1659317	370364	37676	1382
*Medionidus acutissimus*	Mantle lure	Darter/Sculpin	Paul Johnson	306262	1851620	349715	41256	475
*Medionidus conradicus*	Mantle lure	Darter/Sculpin	UF	438914	7718202	1466030	66764	619
*Medionidus parvulus*	Mantle lure	Darter/Sculpin	Paul Johnson	306263	6651085	2082691	62803	604
*Medionidus penicillatus*	Mantle lure	Darter/Sculpin	Paul Johnson	306264	7915534	2253442	80037	660
*Medionidus simpsonianus*	Mantle lure	Darter/Sculpin	Paul Johnson	306265	4362329	1066797	57543	583
*Medionidus walkeri*	Mantle lure	Darter/Sculpin	Paul Johnson	306266	3139933	559539	49255	559
*Obovaria choctawensis*	Mantle lure	Darter/Sculpin	UF	441237	1470462	373459	32610	1052
*Obovaria subrotunda*	Mantle lure	Darter/Sculpin	UF	438391	1672141	601899	32020	1157
*Potamilus ohiensis*	Mantle lure and Simple brood lure	Drum	UF	438806	2251207	785191	34220	294
*Ptychobranchus fasciolarus*	Complex brood lure	Darter/Sculpin	UF	438254	2517640	878247	37577	454
*Ptychobranchus foremanianus*	Complex brood lure	Darter/Sculpin	Paul Johnson	306267	14377252	3961795	78567	659
*Ptychobranchus jonesi*	Complex brood lure	Darter/Sculpin	UF	441272	1455454	491977	29992	355
*Quadrula quadrula*	Mantle lure	Catfish	UF	438787	4999562	1569250	58525	148
*Sagittunio nasutus*	Mantle lure and Simple brood lure	Sunfish and Perch	UF	438285	4608659	1458774	55120	1513
*Sagittunio subrostratus*	Mantle lure and Simple brood lure	Sunfish	UF	441304	1814864	583195	30748	998
*Toxolasma corvunculus*	Mantle lure	Sunfish	UF	440843	2924381	1001628	49472	275
*Toxolasma cylindrellus*	Mantle lure	Sunfish	INHS	49319	11371070	3006669	82040	361
*Toxolasma lividum*	Mantle lure	Sunfish	UF	438185	779097	307476	16824	113
*Toxolasma texasiensis*	Mantle lure	Sunfish	UF	438567	1298761	409308	26318	139
*Truncilla macrodon*	Broadcast	Drum	UF	441301	685468	174606	18594	109
*Truncilla truncata*	Mantle lure	Drum	UF	438976	950716	250143	25987	303
*Venustaconcha ellipsiformis*	Mantle lure and Simple brood lure	Darter/Sculpin	INHS	87179	4434860	1022209	62605	1702
*Venustaconcha trabalis*	Mantle lure and Simple brood lure	Darter/Sculpin	UF	438909	1660491	264191	36956	1469
*Villosa amygdala*	Mantle lure and Simple brood lure	unknown	UF	441054	2021257	400560	39674	1133
*Villosa delumbis*	Mantle lure and Simple brood lure	Bass	UF	437984	4433617	1358358	61582	1544
*Villosa vibex*	Mantle lure and Simple brood lure	Sunfish	UF	438545	1272879	370119	28877	941
*Villosa villosa*	Mantle lure and Simple brood lure	Bass/Sunfish	UF	441268	2756754	671290	48066	1340

**Notes.**

UF University of Florida INHS Illinois Natural History Survey NCS North Carolina State University

Newly sampled material Museum ID refers to their deposition in the UMMZ, University of Michigan Museum of Zoology.

Among the Zanatta & Murphy (2006) taxa that we were unable to source was the genus *Popenaias*, that positioned within the Amblemini in mitochondrial gene trees (Campbell et al., 2005; Zanatta & Murphy, 2006). However more recent studies, using data from the large nuclear ribosomal gene in addition to mt sequences (Pfeiffer et al., 2019), and from an anchored hybrid phylogenomic approach (Pfeiffer, Breinholt & Page, 2019) recovered this genus as members of a newly recognized Mesoamerican and Rio Grande clade, Popenaiadini, sister to Lampsilini.

A non-lethal biopsy technique developed by Berg et al. (1995) was used to collect tissue samples from mussels in the field. Mussel species were categorized based on presence or absence of mantle lure and type of brood lure (simple, complex, or tethered). Mantle lures and brood lures were treated as separate variables because they are not mutually exclusive with many species having both mantle lures and brood lures. The wide spectrum of mantle lure phenotypes found across the clade (Barnhart, Haag & Roston, 2008; Haag, 2012) complicated discrete sub-categorization so this variable was scored simply into presence or absence states. Brood lures were broken down into four categories: absence of brood lure, simple/fragile brood lure, complex brood lure, and tethered brood lure. Information regarding primary hosts, and host infection strategies, for each mussel species (Table 1) was compiled from various literature sources. Reference literature used for each species listed and cited in Table S1.

### ddRADseq data collection and bioinformatics

Genomic DNA was extracted from tissue samples using the E.Z.N.A. Mollusk DNA kit (Omega Bio-Tek, Norcross, GA) according to manufacturer’s instructions and then stored at −80 °C. The quality and quantity of DNA extractions were assessed using a Qubit 2.0 Fluorometer (Life Technologies, Carlsbad, CA) and ddRADseq libraries were prepared following the protocols of Peterson et al. (2012). We then used 200 ng of DNA for each library prep. This involved digestion with Eco-RI-HF and MseI (New England Biolabs, Ipswich, MA) restriction enzymes, followed by isolating 294–394 bp fragments using a Pippen Prep (Sage Science, Beverly, MA) following the manufacturer’s instructions. Prepared ddRADseq libraries then were submitted to the University of Michigan’s DNA sequencing core and run in three different lanes using 150 bp paired-end sequencing on an Illumina HiSeq 2500. Two control individuals of *Lampsilis fasciola* were run in each lane and reads for both individuals clustered together in every analysis with 100% bootstrap support, indicating no lane effects on clustering across individuals. Raw demultiplexed data were deposited at genbank under the bioproject ID PRJNA704566 with accession numbers SAMN18093783–SAMN18093865.

The alignment-clustering algorithm in ipyrad v.0.7.17 (Eaton, 2014; Eaton & Overcast, 2020) was used to identify homologous ddRADseq tags. Ipyrad is capable of detecting insertions and deletions among homologous loci which increases the number of loci recovered at deeper evolutionary scales compared to alternative methods of genomic clustering (Eaton, 2014). Demultiplexing was performed by sorting sequences by barcode, allowing for zero barcode mismatches (parameter 15 setting 0) and a maximum of five low-quality bases (parameter 9). Restriction sites, barcodes, and Illumina adapters were trimmed from the raw sequence reads (parameter 16 setting 2) and bases with low-quality scores (Phred-score < 20, parameter 10 setting 33) were replaced with an N designation. Sequences were discarded if they contained more than 5 N’s (parameter 19). Reads were clustered and aligned within each sample at two different similarity thresholds, 85 and 90% and clusters with a depth <6 were discarded (parameters 11 and 12). We also varied the number of individuals required to share a locus from ∼25% (*N* = 27) to ∼46% (*N* = 50). Ipyrad output files were used for further downstream analyses and are available on Dryad at the following DOI: https://doi.org/10.5061/dryad.c866t1g62.

### Phylogenomic analyses

We analyzed the four concatenated ddRAD-seq alignment files (85% and 90% clustering similarity and 25% and 46% minimum samples per locus) using maximum likelihood in RAxML v8.2.8 (Stamatakis, 2014). A general time-reversible model (Lanave et al., 1984) was used for these analyses that included invariable sites and assumed a gamma distribution. Support was determined for each node using 100 fast parametric bootstrap replications. Due to the relatively deep phylogenetic scale comprised by our taxon sampling, we recovered many more loci with a minimum of 25% individuals per locus and 85% clustering threshold (4,725 loci) compared to runs that included 46% individuals per locus at the same clustering threshold (664 loci). The 90% clustering threshold produced even fewer loci and was not very useful for our phylogenomic analyses. Relationships were robust for most nodes with the 85% clustering threshold, and downstream analyses were performed using both of these datasets (85%–25% and 85%–46%).

The maximum likelihood phylogeny output from RAxML was trimmed to remove the outgroup taxa (*Quadrula quadrula, Amblema plicata, Fusconaia flava,* and *Eurynia dilata*) as well as all multiples of each species using the ‘ape’ package in R version 3.5.2 (R Core Team, 2018; Paradis & Schliep, 2019). This tree with a single individual of each species was used to create an ultrametric tree with two comparable methods using penalized maximum likelihood approaches (Sanderson, 2002; Kim & Sanderson, 2008); one implemented in R using the ‘ape’ package with a correlated rate model (Paradis & Schliep, 2019), and another using treePL (Smith & O’Meara, 2012).

### Ancestral state reconstruction

We analyzed the evolution of mantle lures and brood lures separately because these host infection strategies are neither homologous characters, nor mutually exclusive with many species using both mantle lures and brood lures (Corey, Dowling & Strayer, 2006; Barnhart, Haag & Roston, 2008). For each species of mussel, we independently assessed the mantle lure and brood lure characters and categorized them into binary, present or absent, character states based on the current available data (Table S1). Ancestral State reconstructions for both mantle lures and brood lures were performed using the rerooting method (Yang, Kumar & Nei, 1995), implemented in the ‘Phytools’ package in R (Revell, 2012; Paradis & Schliep, 2019), and using both a one-rate model (ER; equal transition rates among all character states) and a symmetric model (SYM; rates can vary among different traits but forward and reverse transition are constrained) where rates are allowed to differ between transitions but are constrained between forward and reverse transitions.

### Lampsiline diversification rates

Two different approaches were used to investigate the potential influence of host infection strategies on diversification rates in the Lampsilini. The first method used State Speciation and Extinction models to explicitly test the association between host infection strategies and diversification rates, the second method used BAMM to estimate diversification rates and evidence of rate shifts in the lampsiline phylogeny

Hidden State Speciation and Extinction models were implemented using the ‘hisse’ package in R (Beaulieu & O’Meara, 2016). Four models were performed independently for each trait (presence of mantle lure, presence of brood lure, broadcast release); a binary state-dependent model (BiSSE), a hidden state dependent model (HiSSE), a two-state character-independent model, and a four-state character independent model. The two-state and four state character-independent models were included as null models to compare to the BiSSE and HiSSE models. Rabosky & Goldberg (2015) found that BiSSE models tend to have a high type-1 error rate when compared to a null model that assumes homogenous diversification rates across the tree. The two-state and four-state character-independent models were proposed as an alternative null model which allows for rates to vary, independent of the trait value, and reduces type-1 error rates (Beaulieu & O’Meara, 2016). All models allowed extinction rates to vary independently for each character state, and transition rates between states were fixed to simplify the models. The revised freshwater mussel taxonomy by Williams et al. (2017) was used to estimate sampling frequency for each trait category. This analysis was performed with both the ultrametric tree derived from the ‘ape’ package as well as the one derived using TreePL. To further explore state-dependent models, the R package ‘Diversitree’ was used to estimate and visualize diversification rates using an MCMC approach (FitzJohn, 2012).

For the second method, we used Bayesian Analysis of Macroevolutionary Mixtures (BAMM) software package (v. 2.5) and the R package “BAMMtools” to estimate diversification rates in the Lampsilini phylogeny, (Rabosky, 2014; Rabosky et al., 2014). BAMM uses a reversible-jump Markov Chain Monte Carlo to automatically detect clades that share common evolutionary parameters of diversification (Rabosky et al., 2013). BAMM was performed using 10,000,000 generations, sampling every 5,000 generations. Priors for the model were selected using the setBAMMpriors function in R (Rabosky et al., 2014). To account for incomplete taxon sampling, we used previously published mitochondrial phylogenies for this group (Campbell et al., 2005; Zanatta & Murphy, 2006) and the revised list of freshwater mussels of the United States and Canada by Williams et al. (2017) to estimate clade-specific frequencies of sampling biases.

## Results

### ddRADseq data collection and bioinformatics

Illumina sequencing returned raw reads ranging from 287,978 to 14,377,252 per individual across the 83 unionid samples included in the analyses. Mean coverage depth, for the 85% clustering threshold, ranged from 1.48 (*Toxolasma lividum*) to 5.25 (*Lampsilis virescens*) (Table 1, Table S2).

We identified between 4,745 and 664 homologous loci across the two best ddrad datasets (85%–25% and 85%–46%) and, in general, much higher numbers of loci were recovered for the core Lampsilini ingroup (>1,000 loci) relative to the outgroups (<100 loci). Although lowering clustering thresholds produced a much greater amount of missing data in the ddrad supermatrix, they also greatly increased the number of loci which could be used, *e.g*., for the 85% clustering threshold, 664 loci were recovered when a minimum of 46% individuals were included, whereas a 25% minimum yielded 4,745 loci. Simulation studies and empirical analyses both suggest that large amounts of missing data may be relatively unproblematic for phylogenetic reconstructions, especially if the total dataset is large (Rubin, Ree & Moreau, 2012; Huang & Knowles, 2016; Eaton et al., 2016). Datasets recovered from both the 25% and 46% minimum samples per locus clustering thresholds were used in all our phylogenomic analyses.

### Phylogenomic analyses

**Figure 3:**
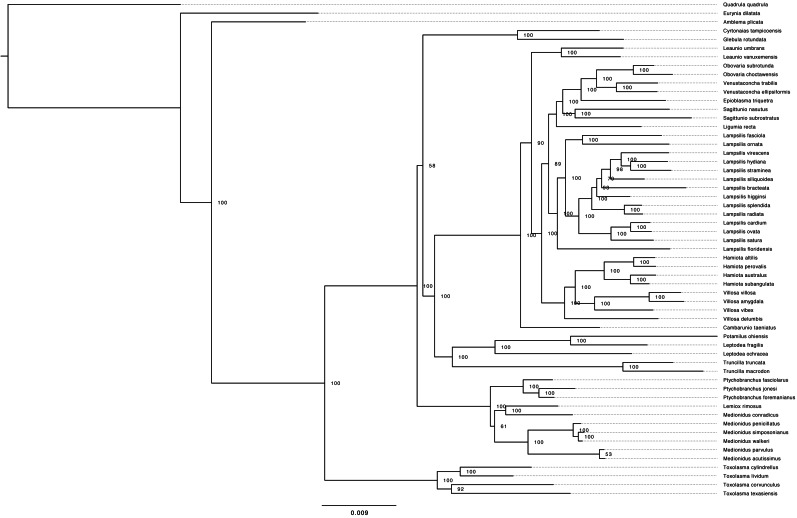
Phylogeny of lampsiline mussels. Maximum likelihood phylogeny of North American lampsiline mussels created with RAxML v8.2.8 using a general time reversible model from the 85% clustering threshold with 25% minimum samples per locus dataset. Support for each node was determined using 100 fast parametric bootstrap replications. Bootstrap values are adjacent to each node. Scale bar represents mean number of base pair substitutions per site.

The ddRADseq gene tree topologies we recovered were highly consistent across all of the parameter settings analyzed, with a few differences in placement of poorly supported nodes (Fig. 3 and Fig. S1). All our phylogenetic trees recovered the monophyletic genus *Toxolasma* as sister to the other members of the Lampsilini tribe included in the study. The latter formed four well-supported crown clades, each composed of members of >1 genus: a 2-species clade with *Glebula* and *Cytronaias* spp., a 10-species clade with *Medionidus*, *Lemiox*, and *Pytchobranchus* spp., a 5-species clade containing *Leptodea*, *Potamilus* and *Truncilla* spp., and a 33-species clade containing *Ligumia*, *Epioblasma*, *Obovaria*, *Venustaconcha*, *Hamiota*, *Villosa, Sagittunio, Cambarunio, Leaunio* and *Lampsilis* spp. Across our topologies, some genera were recovered as monophyletic (*Toxoplasma* (4 species), *Obovaria* (2 species), *Venustaconcha* (2 species) *Hamiota* (4 species), *Lampsilis* (14 species), *Pytchobranchus* (3 species), *Sagittunio* (2 species; see Watters, 2018), *Leaunio* (2 species; see Watters, 2018), but some others did not (*Medionidus* (6 species), *Leptodea* (2 species)). The new reclassification of *Villosa* suggested by Watters (2018) is supported in our analyses for the species we have included.

**Figure 4:**
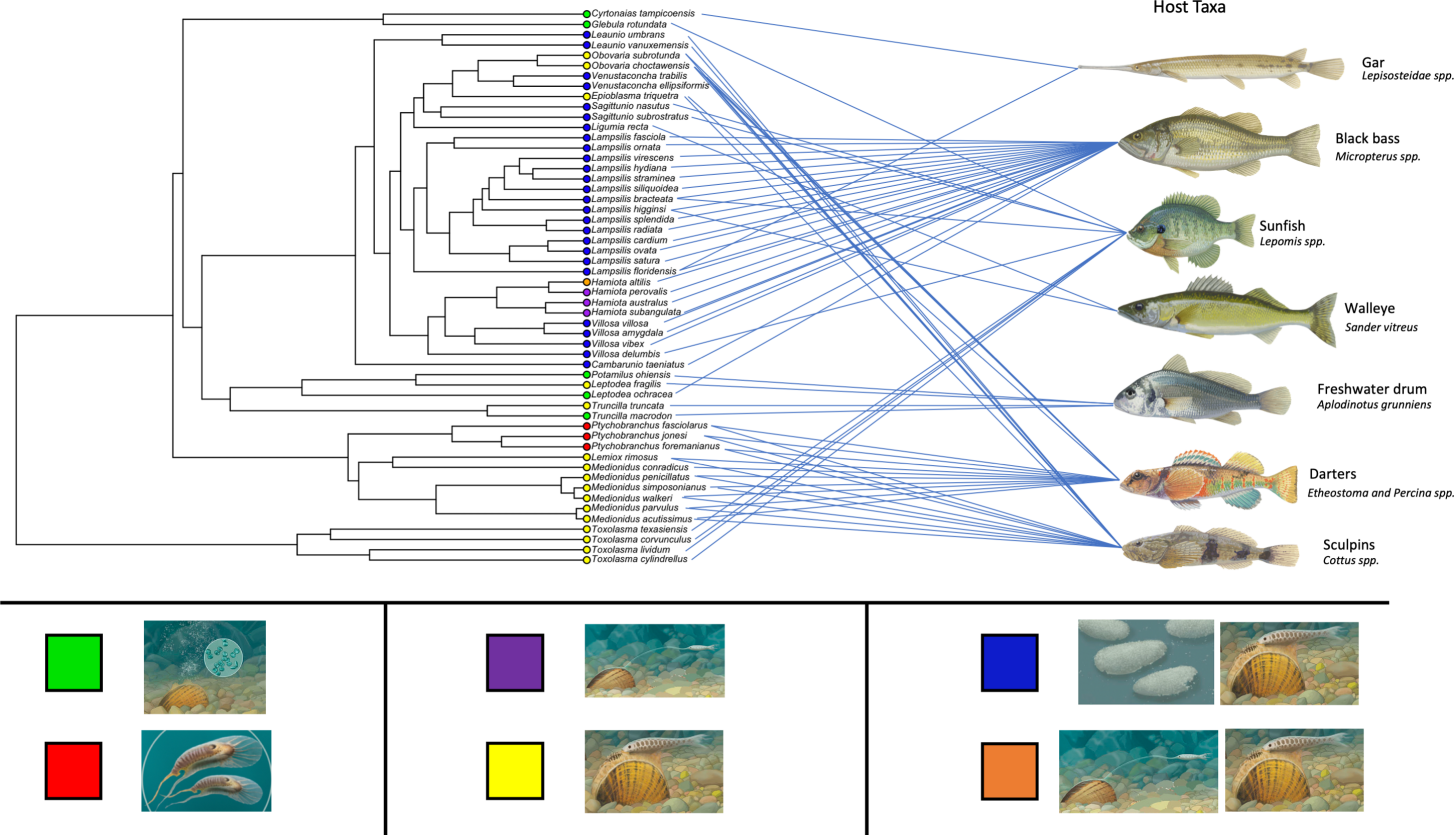
Freshwater mussel phylogeny including host infection strategy and preferred hosts for each species. Ultrametric phylogeny created from maximum likelihood phylogeny of lampsiline mussels (Fig. 4) using TreePL. This tree was trimmed to remove outgroups and retain only a single individual per species. Tips are color coded based on the known host infection strategies used by each species: green = broadcast. red = complex brood lure, purple = tethered brood lure, yellow = mantle lure, blue = mantle lure and simple brood lure, and orange = mantle lure and tethered brood lure. See Barnhart, Haag & Roston (2008) for an in-depth review of host infection strategies. Primary host type for each mussel species is visualized by connecting lines. Sources used for determining host use are found in Table S1. Fish and host infection strategy illustrations by John Megahan.

An ultrametic tree (Fig. 4) was created with TreePL from the 85% clustering similarity with 25% minimum samples per locus topology (Fig. 3) and manually pruned to one individual per species according to read count. The mussel species are color-coded according to their host infection strategy and their primary (most frequently used) host taxa are indicated. A striking feature of this topology is the high degree of conservation shown by ingroup mussels in their primary fish host taxa, *e.g.*, the mantle-lure producing *Toxolasma* spp. clade with sunfishes (*Lepomis* spp.), the mixed strategy dominated 33-species clade primarily with bass (*Micropterus* spp.), the mantle lure or brood lure 10-species *Medionidus*/*Lemiox*/*Ptychobranchus* spp. clade with darters (*Etheostoma* spp.) and sculpins (*Cottus* spp.), and the 5-species *Leptodea*/*Potamilus*/*Truncilla* spp. clade–some broadcasting larvae, some with mantle lures–with freshwater drum (*Aplodinotus grunniens*).

### Ancestral state reconstruction

**Figure 5:**
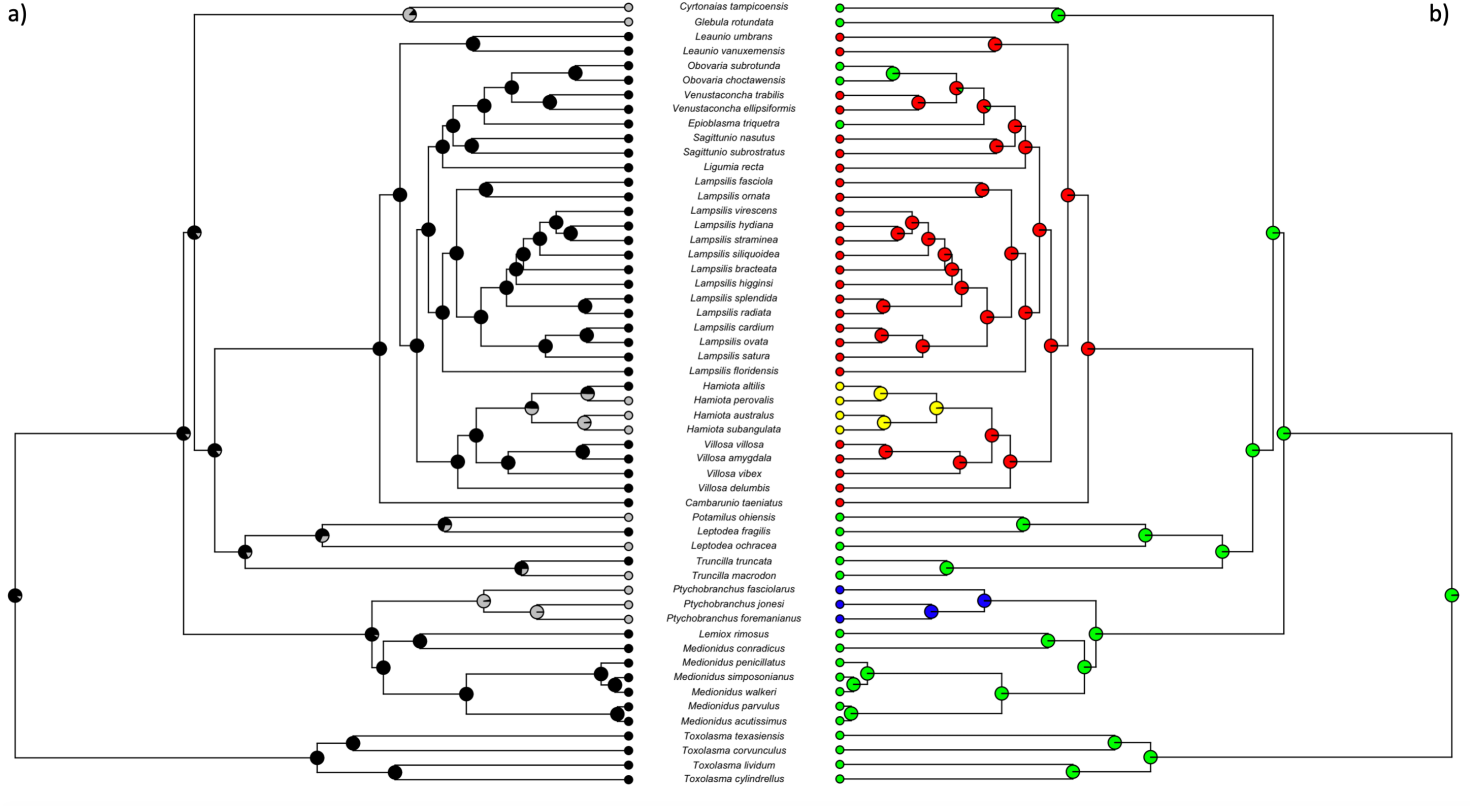
Ancestral state reconstructions of mantle lures and brood lures in lampsiline mussels. Ultrametric phylogenies created from maximum likelihood phylogeny of lampsiline mussels (Fig. 3) using TreePL. These trees were trimmed to remove outgroups and retain only a single individual per species. (A) Ancestral state reconstruction of mantle lures using a symmetrical rates model: Black = presence of a mantle lure (Fig. 2D), Grey = no mantle lure. (B) Ancestral state reconstruction of brood lures using a symmetrical rates model: Blue = complex brood lure (Fig. 2F), Red = simple brood lure (Fig. 2E), Yellow = complex tethered brood lure (Fig. 2H), Green = no brood lure.

Ancestral state reconstructions were performed for both mantle lures and for brood lures using two different models for transition rates (ER and SYM). The likelihood values for the mantle lure models are ER = −24.65 and SYM = −24.65. For brood lure reconstructions the likelihood values are ER = −26.14 and SYM = −23.81. Using the SYM model, estimated probabilities of character states at each node were plotted on the ultrametric tree (Fig. 5; Fig. S2). These results imply that mantle lures evolved early in the Lampsilini phylogeny, being present in the ingroup’s last common ancestor, with four to six subsequent losses. Brood lures are inferred to have independently evolved twice in this phylogeny.

Gain of a complex brood lure was coupled with loss of a mantle lure in *Pytchobranchus* (Fig. 5), although this transition was not associated with a change in primary host fishes (darters/sculpins; Fig. 4). Gain of a simple brood lure in ancestor of the 33-species, 10-genus, predominantly bass host specialist clade did not result in the loss of a mantle lure. However, within that clade, the subsequent evolution of a complex, tethered brood lure in *Hamiota* was associated with the loss of a mantle lure in 3/4 species (Fig. 5), but no change in primary host fishes (Fig. 4). Eleven of the 33 species in this clade have primary fish hosts other than bass (darters/sculpins (7), sunfishes (3), and walleye (1)) and, while all of them have retained mantle lures, 3 of the 7 species targeting darters/sculpins–*Obovaria subrotunda*, *Obovaria choctawensis* and *Epioblasma triquetra*–have lost simple brood lures, with the latter species physically capturing host fish to enable larval infection (Fig. 2C). The remaining cases of mantle lure loss are associated with the gain of broadcast larval release in two clades: one containing the gar specialist *Cyrtonaias tampicoensis* and the sunfish specialist *Glebula rotundata*, the other involving three members of the 5-species *Leptodea*/*Potamilus*/*Truncilla* spp. clade: the drum (*Aplodinotus grunniens*) specialists *Truncilla macrodon* and *Potamilus ohiensis* and the white perch (*Morone americana*) specialist *Leptodea ochracea* (Figs. 4 and 5).

### Lampsiline diversification rates

**Figure 6:**
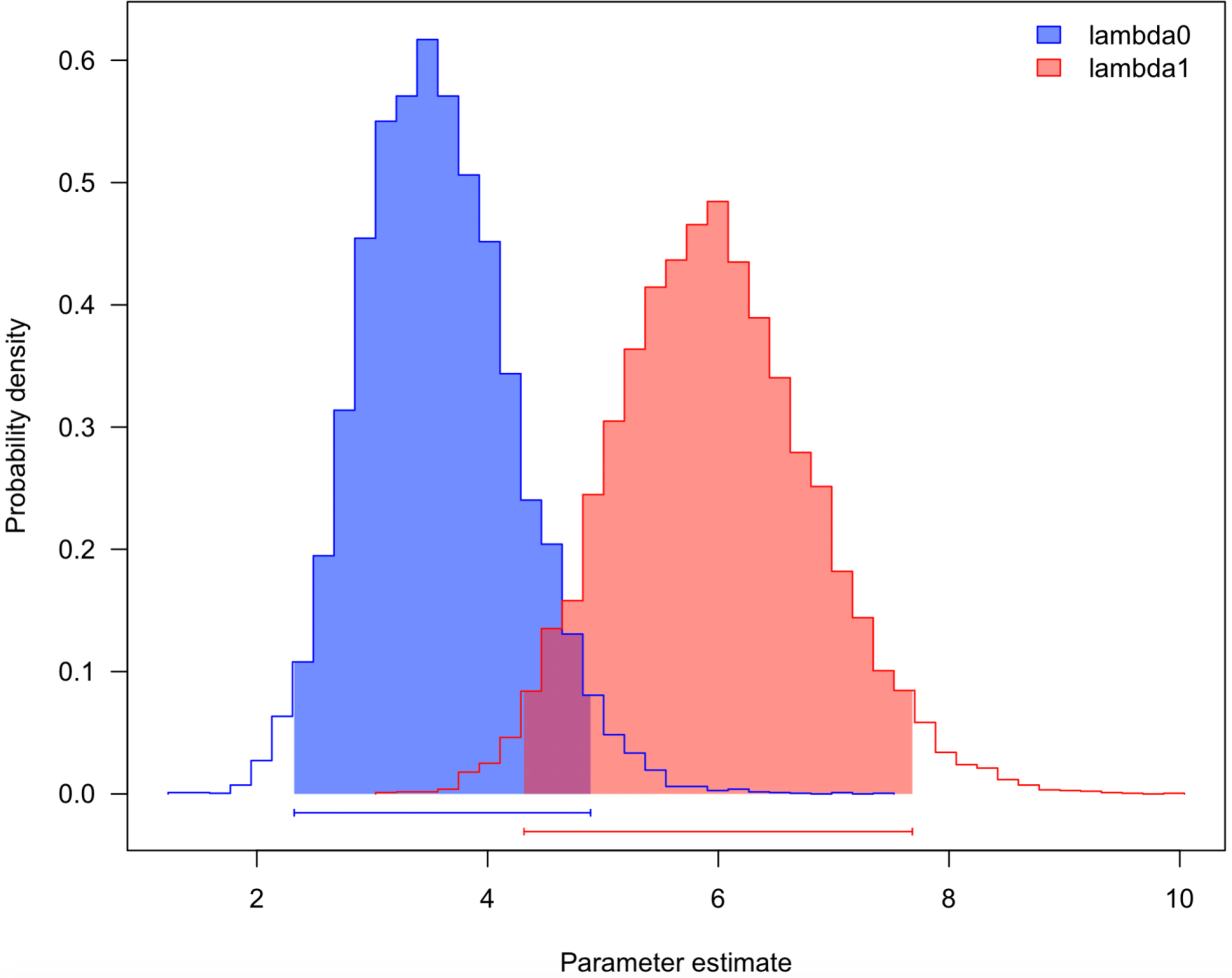
Diversification rate estimates for species with and without brood lures. Parameter estimates for net diversification rates between species without a brood lure (lambda0) and species with a brood lure (lambda1). Parameters were estimated using a MCMC approach, implemented in the R package ‘diversitree’ for 10,000 generations.

Three traits were assessed independently (mantle lure, brood lure, and broadcast release) using four different models (BiSSE, HiSSE, 2 state character independent, and 4 state character independent; Table 2). The best-performing model (AICc) for the mantle lure trait was the two-state independent model, suggesting no relationship between mantle lures and net diversification rates. The BiSSE model was the best-performing model (AICc) for the brood lure trait by a small margin, suggesting an increase in net diversification rate for species with brood lures (estimated net diversification rate of 11.7 for species with brood lure versus 8.5 for those without) and largely similar estimates for extinction fraction, which is the ratio of extinction rate/speciation rate (0.38 versus 0.41 respectively). This result was consistent across both the 25% minimum samples per locus topology (Table 2) and the 46% topology (Table S3), regardless of how the ultrametric tree was derived. To explore these models further, we used an MCMC modeling approach, implemented in the R package ‘diversitree’ (FitzJohn, 2012) to estimate diversification rates for species with and without brood lures. The distributions for the parameter estimates have some overlap (Fig. 6) but display two distinct peaks and the species with brood lures have a higher estimated diversification rate. When analyzing the 85%–46% tree (Fig. S1), we found the BiSSE model was also the best-performing model (AICc) for broadcast release by a small margin (Table S3), hinting at a possible reduced diversification rate for broadcast releasers, but this was result was not corroborated in the 85%–25% tree (Table 2).

**Table 2:** AIC and log likelihood values for SSE models performed for three different life history traits. Displays the AIC, AICc, and log likelihood values for a set of state dependent speciation models performed independently for three different traits: Mantle lure, Brood lure, and broadcast strategy. The four models performed for each trait include a BiSSE model (2 state trait dependent), a HiSSE model (4 state model with two trait states and two hidden states), a 2-state trait independent null model, and a 4 state trait independent null model.

	Mantle lure			Brood lure			Broadcast strategy		
Model name	AIC	AICc	Log likelihood	AIC	AICc	Log likelihood	AIC	AICc	Log likelihood
2-state CID	**24.88**	**26.13**	**−7.4420**	16.01	17.25	−3.0014	**5.43**	**6.68**	**2.2836**
BiSSE	30.35	31.60	−10.1743	**8.59**	**9.84**	**0.7047**	8.42	9.67	0.7899
4-state CID	30.15	34.24	−6.0748	17.03	21.12	0.4830	11.16	15.26	3.4179
HiSSE	33.74	37.83	−7.8684	16.45	20.54	0.7739	13.85	17.94	2.0739

**Figure 7:**
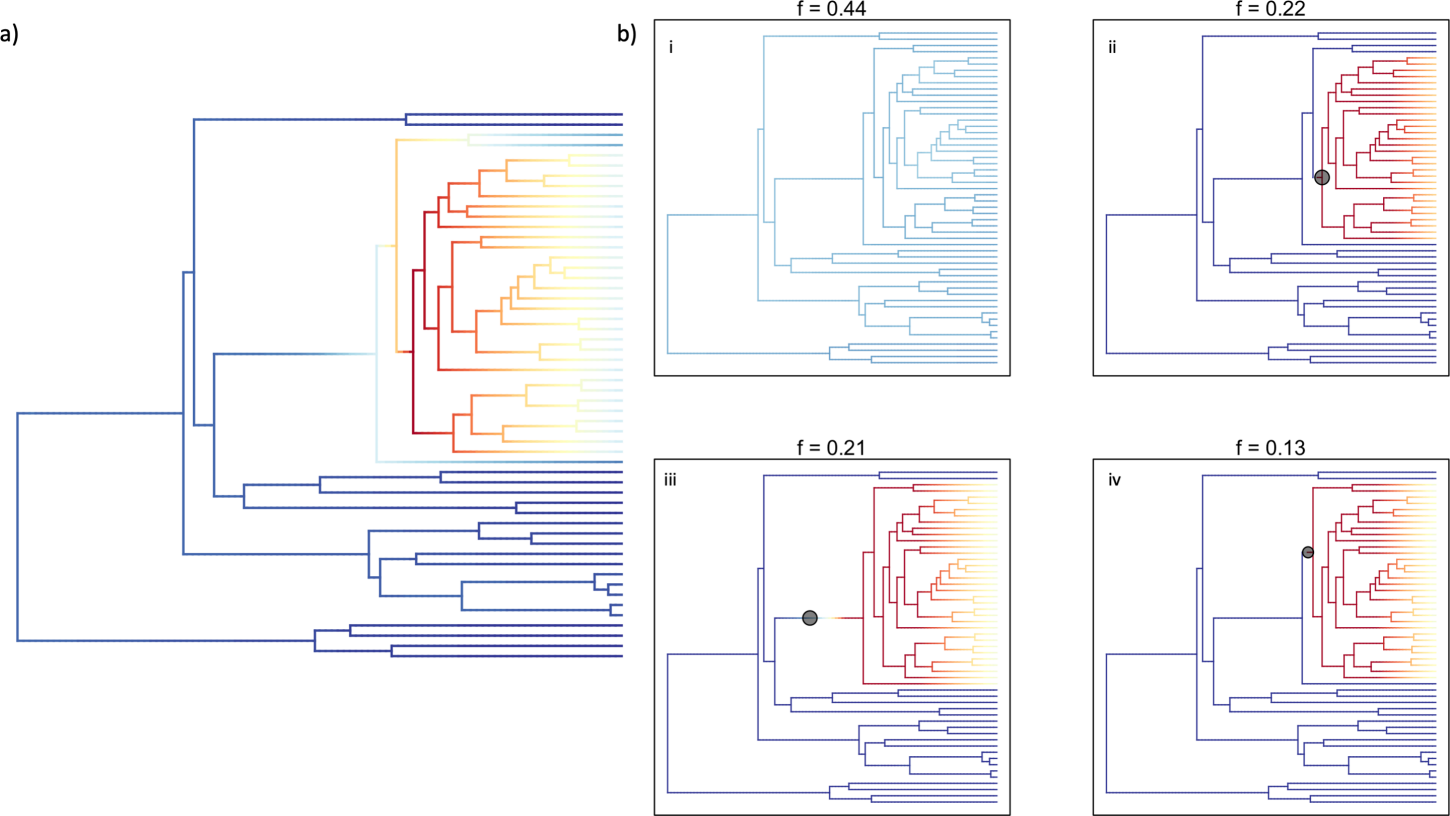
Most credible rate regimes estimated by BAMM for lampsiline mussels. BAMM analyses results showing the average modeled rate shifts per each branch (A) and the most credible shift sets (B) generated for the Lampsilini ingroup. The four trees shown in B (I–IV) represent the four most frequently sampled rate regimes sampled by BAMM and their respective frequencies are displayed above each tree.

We tested for differences in speciation rates among the 54 species of lampsilines by performing BAMM analyses for 10,000,000 generations on the ultrametric tree (Fig. 4). The mean, model averaged diversification rates estimated along each branch are displayed in Fig. 7A and all four credible rate-shift sets recovered are displayed in Fig. 7B. The best rate-shift configuration (*f* = 0.44) suggests a static diversification rate across the entire ingroup topology, with no clade-specific differences in diversification rate (Fig. 7A). However, the second, third and fourth most sampled rate-shift configurations (*f* = 0.22, 0.21 and 0.13), comprising 56% of configurations sampled, indicate an increase in diversification rate on adjacent stem branches of the 33-species clade containing *Ligumia*, *Epioblasma*, *Obovaria*, *Venustaconcha*, *Hamiota*, *Villosa, Sagittunio, Cambarunio, Leaunio,* and *Lampsilis* spp. (Figs. 7Bii-7Biv).

## Discussion

### Evolution of infection strategies in lampsiline mussels

Our genomic phylogeny of Lampsilini represents a robust and comprehensive inferred evolutionary history of this North American unionid tribe. In contrast with earlier mitochondrial phylogenies (Campbell et al., 2005; Zanatta & Murphy, 2006), nodal support throughout the topology (Fig. 3) was generally high: a large majority of nodes displayed support values of 100 and only 15% had values <90. Most of the latter were concentrated within the *Lampsilis* clade, with the exception of the placement of the *Villosa* and *Hamiota* clade, and may stem from either incomplete lineage sorting or hybridization processes (Maddison & Knowles, 2006), but this question requires further investigation. Nevertheless, it is important to emphasize that our genomic phylogeny agrees broadly with those of previous molecular studies both in regard to outgroup/ingroup (Campbell et al., 2005) and among-ingroup (Campbell et al., 2005; Zanatta & Murphy, 2006; Pfeiffer, Breinholt & Page, 2019) relationships.

Our phylogenomic analyses (Fig. 4) indicate that fish host use in the Lampsilini through time is characterized by a high degree of mussel clade specificity for both primary host type and host infection mechanism(s). This result corroborates Haag’s (2012) suggestion that host use is highly conserved in this group as well as Hewitt, Wood & Foighil’s (2019) finding of topological congruence between North American unionids and their hosts. It also implies that lure-based host infection mechanisms are adaptive in origin, being specialized for attracting suitable hosts, as has been observed in the wild for a subset of co-occurring mussels (Haag & Warren, 2003). There are numerous examples of such across our tree topology (Fig. 4), *e.g.*, most *Lampsilis* species target bass (*Micropterus* spp. - predators that are highly piscivorous when large (Hickley et al., 1994)) as primary hosts using large, conspicuous mantle lures that typically resemble small fishes (Barnhart, Haag & Roston, 2008). Likewise, *Toxolasma* species have a worm-like mantle lure (Fig. 2ii) and predominantly target sunfishes in the genus *Lepomis* that are generalist predators with a diet that includes worms (Parsons & Robinson, 2007). Finally, the clade composed of *Medionidus* spp. (with small, cryptic mantle lures) and *Ptychobranchus* spp. (with small demersal brood lures that typically mimic insect or fish larvae) specialize in darters and sculpins (small, benthic predatory fishes (Haag, 2012; Cummings & Watters, 2017)).

Our ancestral state reconstruction results corroborated Graf & Ó Foighil’s (2000) and Zanatta & Murphy’s (2006) mt phylogeny-based inferences that lampsiline mantle lures evolved early in this clade, followed by multiple secondary losses. These inferred losses occurred across much of the ingroup topology, apart for the genus *Toxolasma* (characterized by its worm-like mantle lures), and mantle lure loss was associated with the *de novo* gain of either complex brood lures or of broadcast infection strategies (Fig. 5; Fig. S2). The former occurred independently in two genera [*Ptychobranchus*, and in 3/4 of the *Hamiota* species represented] and involved a change in mimetic lure type: from mimetic mantle lures to mimetic brood lures, although *Hamiota altilis* retains both. The latter cases of mantle lure loss, inferred separately for *Cyrtonaias tampicoensis* and *Glebula rotundata*, and for *Leptodea ochracea, Potamilus ohiensis* and *Truncilla macrodon*, were more radical in that they involved the abandonment of prey mimicry and host deception as a host infection strategy. Haag & Warren (1998) found that population densities of specialist mussels and their fish hosts were correlated for broadcasters, but not so for lure-producing mussels. The evolutionary loss of lures in host specialist mussels would therefore appear counterintuitive, especially for mussels with low-density fish hosts, but there are potentially mitigating life history traits in some of these taxa that may act to increase their rate of host infection.

One such life history trait is increased larval production: relative to other lampsilines, *Glebula rotundata* females release more larval broods per year (Parker, Hackney & Vidrine, 1984) and the genera *Truncilla* and *Leptodea* have higher fecundities and smaller-sized larvae (Haag, 2013). Another such trait may involve targeting mussel predators as larval hosts, *e.g.*, adult *Aplodinotus grunniens* (freshwater drum) prey on mussels and at least some of the species that use it as a host may engage in a sacrificial strategy whereby infection occurs when gravid females (especially smaller specimens) are consumed (Barnhart, Haag & Roston, 2008; Haag, 2012). Four of five members of the *Leptodea*/*Potamilus*/*Truncilla* spp. clade (Fig. 4) are *A*. *arunniens* specialists [the fifth, *L. ochracea*, occurs outside of this fish’s range (Page & Burr, 2011)] and, until recently, it was assumed that these three mussel genera lacked mantle lures. However, Sietman, Hove & Davis (2018) documented the presence of cryptic, nocturnally displayed, mantle lures for one member of each of these genera (including *Truncilla truncata* and *Leptodea fragilis*). In light of these new data, we view the current categorization of *Leptodea ochracea, Potamilus ohiensis* and *Truncilla macrodon* as lacking mantle lures (Figs. 4 and 5) to be provisional. For the taxa included here, our topology corroborates that of Smith, Pfeiffer & Johnson (2020) and our data support their decision to reclassify *Leptodea ochracea* to *Atlanticoncha ochracea*.

Our ancestral state reconstruction of brood lures (Fig. 5B) is consistent with two origins (one each in the genera *Ptychobranchus* and *Hamiota*) of complex, mimetic brood lures, and one additional origin of simple, non-mimetic brood lures in the ancestor of the 33-species, 10-genus, predominantly bass host specialist clade (Fig. 5B). The latter clade contains *Hamiota*, implying that the complex tethered brood lure found in *Hamiota* species (Fig. 2H) may be derived from the simple brood lures found in most of this clade, including species of its sister genus *Villosa* (Fig. 5B). In contrast, the darter/sculpin specialist clade containing *Ptychobranchus* (Fig. 4) lacks simple, non-mimetic brood lures (Fig. 5B). The evolutionary origins of the *Ptychobranchus* demersal mimetic brood lure (Fig. 2F) may stem from a common ancestor with the genus *Cyprogenia*. Previous mt phylogenies (Campbell et al., 2005; Zanatta & Murphy, 2006) have placed the genus *Cyprogenia*, with its demersal, mimetic baited brood lures (Barnhart, Haag & Roston, 2008), sister to the genus *Ptychobranchus*. Unfortunately, we failed to extract sufficient genomic data for our *Cyprogenia stegaria* sample to corroborate this relationship.

The 10-genus, predominantly bass host specialist clade comprised 33 species (Fig. 4) of which 26 (in the genera *Lampsilis, Villosa, Ligumia, Leaunio, Cambarunio, Sagittunio,* and *Venustaconcha*) produce mantle lures as well as simple brood lures (Figs. 5A, 5B). Mantle lures are regarded as their primary method of infecting fish hosts (Haag & Warren, 2000; Barnhart, Haag & Roston, 2008; Gascho Landis et al., 2012) and a gravid female may display hers for weeks to months (Kraemer, 1970; Haag & Warren, 2003). During an elicited host fish attack on mantle lure-displaying *Lampsilis* spp. gravid females, glochidia are extracted (Barnhart, Haag & Roston, 2008) from only a subset of their ∼60 marsupium water tubes and displaying females often exhibit a mix of undischarged (*i.e.*, containing larvae) and discharged water tubes for much of the spring/summer host infection season (Haag & Warren, 1999). Lampsiline mussels have evolved bradytictic life cycles in which spawning typically occurs in the late summer and the resulting larvae are brooded overwinter (Graf & Ó Foighil, 2000). Gravid females must therefore release the previous year’s brood to facilitate fertilization and retention of their new clutch of eggs and it was initially unclear if the release of simple brood lures in these species represented a default end-season emptying of marsupial water tubes (Barnhart, Haag & Roston, 2008), a stress response to captivity (Corey, Dowling & Strayer, 2006), or a supplementary host infection strategy (Corey, Dowling & Strayer, 2006; Barnhart, Haag & Roston, 2008; Haag, 2012). Gascho Landis et al. (2012) performed a detailed experimental study of mantle lure display and simple brood lure production in *Ligumia subrostrata* and concluded that the latter clearly represents a secondary bet-hedging infection strategy. Nevertheless, the relative attractiveness of simple brood lures as putative food items to host fishes remains to be established as does their durability in nature: they typically break up quickly after release (Barnhart, Haag & Roston, 2008).

**Figure 8:**
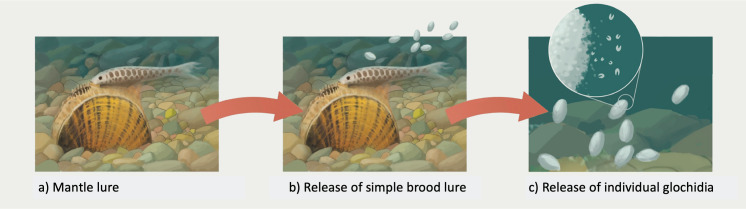
Illustration of a hypothesized three-step bet-hedging strategy. A three-step generalized hypothetical bet-hedging host infection strategy for gravid mussels that produce both a mantle lure and a simple brood lure (genera *Lampsilis, Ligumia, Venustaconcha, Sagittunio, Leaunio, Cambarunio,* and *Villosa*). The first two steps are based on Gascho Landis et al. (2012) and consist of a prolonged mantle lure display (A, the primary strategy) for much of the host infection season, followed by release of residual brooded larvae later in the season within simple brood lures (B, the secondary strategy). Most simple brood lures are fragile and quickly break up releasing larvae (Barnhart, Haag & Roston, 2008). We propose that this latter process represents a tertiary larval broadcast strategy (C) that may occur in more lotic habitats where water movement is sufficient to keep individual larvae in suspension. Illustrations by John Megahan.

Based on available data, we propose a hypothesized three-step bet-hedging host infection strategy in these mussels (Fig. 8). This would involve (A) host attraction and infection via prolonged maternal mantle lure display; (B) the secondary release of residual brooded larvae within simple brood lures prior to the onset of seasonal spawning; and (C) tertiary broadcast dispersal (in lotic habitats) of individual infective larvae following simple brood lure breakup, although the probability of broadcast larvae encountering a host is likely low (Jansen, Bauer & Zahner-Meike, 2001) unless the latter is locally abundant.

The genus *Epioblasma* is a notable exception to the modal host infection mechanism found in this 10-genus crown clade in that gravid females produce mantle lures only and specialize in darter hosts that they actively trap during the infection process using female-specific shell margin extensions (Barnhart, Haag & Roston, 2008). This genus is highly underrepresented in our study with only one member, *E. triquetra*, included; a shortcoming primarily due to the exceptionally intense extinction pressure the genus has been subjected to over the past century. Of the 28 currently recognized species of *Epioblasma* (Williams et al., 2017), 13 are listed as extinct on the IUCN Red List and most of the remainder are critically endangered.

### Diversification rates

The BAMM and state-dependent speciation model analyses yielded new insights into lampsiline diversification rates albeit with some methodological and sampling (*e.g*., the genus *Epioblasma*) caveats. The most supported BAMM result—a single diversification rate regime across the entire Lampsilini clade (Fig. 7I)—needs to be treated with caution as this methodology is biased towards zero rate shifts in smaller trees that contain fewer than approximately 150 species (Rabosky, Mitchell & Chang, 2017; Kodandaramaiah & Murali, 2018). In contrast, the three next-most supported results (Figs. 7Bii–7Biv) identified inferred rate shift accelerations that were tightly clustered on adjacent stem nodes of the 10-genus/33-species crown clade. This collective topological placement bracketed the inferred origin of the mixed infection strategy predominant in this crown clade that combines the use of mantle lures, a plesiomorphic trait (Fig. 5A), with simple brood lures, a derived trait (Fig. 5B). That topological congruence is broadly consistent with the BiSSE (Table 2; Table S3) and MCMC (Fig. 6) modeling results that found evidence for increasing diversification rates among lampsiline species with brood lures. However, it must be emphasized that the majority of these species produce simple brood lures and are likely to rely on mantle lures as their primary host infection strategy (Gascho Landis et al., 2012). Barnhart, Haag & Roston (2008) suggested that species that use both mantle lures and brood lures (conglutinates) could potentially parasitize both large- and small-bodied hosts (the latter being less likely to attack mantle lures). Similarly, a hypothesized three-step bet-hedging strategy (Fig. 8) could potentially generate higher diversification rates by expanding the repertoire of potential host fishes and thereby decreasing the risk of extinction. However, testing such a hypothesis requires significantly better data on host infection processes in natural populations as well as a more comprehensive phylogeny of unionids. The latter is also required to adequately address another outstanding question: the relative diversification rates of broadcasters and lure-using mussel taxa. It is notable that in a broadly parallel case, the evolution of deceit pollination in orchids apparently did not increase their rate of net diversification (Givnish et al., 2015).

### Adaptive radiation of lampsilini

Models of adaptive radiation predict that the availability of ecological niches within an environment, and the response of adapting lineages to occupy them, drive and modulate this important evolutionary process. (Schluter, 1996; Gavrilets & Losos, 2009; Losos, 2010; Arbour & López-Fernández, 2016). Our primary phylogenomic result—that lampsiline clades are highly specific in primary fish host type and in host infection mechanism—is consistent with adaptive radiation expectations in regard to their larval ecology, despite the relatively brief duration of this life history stage. Two factors may bear on this ostensibly surprising result. Once lampsiline mussels evolved a high degree of fish host specialization (Haag & Warren, 1998), the number of discrete larval ecological niches potentially available to them, in the form of local host fish species diversity, greatly increased. In addition, successful larval infection and metamorphosis (transformation) on a fish host is a necessary precondition for juvenile mussel recruitment, and therefore for ecological persistence, in wild populations.

Although our data support an adaptive radiation framework operating at the level of lampsiline clades, they lack the fine-grained resolution of specific host data needed to establish if it equally applies to within-clade diversification. For instance, it remains to be established to what degree sympatric, closely related lampsiline species preferentially target different species of host within the same host guild, consistent with a seamless adaptive radiation paradigm, or rather compete for the same host species, consistent with an evolutionary arms race paradigm (Van Valen, 1977). We anticipate that the balance of these two potential within-clade evolutionary processes may differ among lampsiline lineages according to the range of potential hosts available to them. For example, there are ∼200 species of North American darters, many with small ranges (Near et al., 2011), and there may be considerable evolutionary scope for a high degree of host exclusivity and within-clade adaptive radiation among the darter-specialist lampsiline genera such as *Medionidus, Ptychobranchus* and *Epioblasma*. In contrast, there are fewer (∼41; Roe, Harris & Mayden, 2002; Baker, Blanton & Johnston, 2013; Freeman et al., 2015) species of centrarchids in North America than of lampsiline centrarchid specialists (∼50; Williams et al., 2017). Although new centrarchids species continue to be described (Baker, Blanton & Johnston, 2013; Freeman et al., 2015), the lower number of potential centrarchid hosts implies that some of these mussel species are more likely to compete directly, when in sympatry, for the same hosts and thereby become entrained in an evolutionary arms race for lure effectiveness. In such cases, coexistence could be modulated by frequency-dependent selection processes (Endler, 1988), in which previously infected host fishes are more likely to engage with unfamiliar/rare lure phenotypes, a process that has also been implicated in the evolution of lure polymorphisms in some lampsiline species (Zanatta, Fraley & Murphy, 2007; Barnhart, Haag & Roston, 2008).

## Conclusions

Unionoida is by far the most speciose freshwater bivalve order (Graf & Cummings, 2007) and this richness was especially heightened in southeastern US watersheds, prior to their destructive 20th century industrialization (Lydeard et al., 2004). A record 69 species—the Muscle Shoals fauna—was recorded in the middle reaches of the Tennessee River (Garner & McGregor, 2001), each of them dependent on successful larval parasitism of fish hosts for their recruitment and survival. There is an emerging consensus among mussel researchers that larval partitioning of ambient fish host resources is common in diverse North American unionoid communities (Barnhart, Haag & Roston, 2008; Haag, 2012; Cummings & Watters, 2017; Hewitt, Wood & Ó Foighil, 2019) and that the presence of discrete larval niches may explain the persistence of species-rich mussel assemblages over ecological timescales (Rashleigh & De Angelis, 2007). We propose that these larval niches are evolutionary end-products of cryptic adaptive radiation processes, operating in these watersheds over long time scales (Losos, 2010; Arbour & López-Fernández, 2016), but we acknowledge that much more detailed field work is required to build a comprehensive understanding of their extent and scope.

##  Supplemental Information

10.7717/peerj.12287/supp-1 Supplemental Information 1 Supplemental Figures and Tables Table S1: Summary of samples used in our analyses including; where samples were obtained, host infection mechanism used, primary host fish, and sources cited for determining host use and host infection mechanisms. NCS = North Carolina State University, UF = University of Florida, INHS = Illinois Natural History Survey, and AABC = Alabama Aquatic Biodiversity Center.
Table S2 : Summary of the final number of ddRAD-seq loci for each individual at the 85% and 90% clustering similarity threshold and for 25% and 46% minimum samples per loci. Table S3: Displays the AIC, AICc, and log likelihood values for a set of state dependent speciation models performed independently for three different traits: Mantle lure, Brood lure, and broadcast strategy. The four models performed for each trait include a BiSSE model (2 state trait dependent), a HiSSE model (4 state model with two trait states and two hidden states), a 2-state trait independent null model, and a 4 state trait independent null model. Analysis Performed with the topology recovered using 85% clustering threshold and 46% minimum samples per locus. Figure. S1: Maximum likelihood phylogeny of North American lampsiline mussels created with RAxML v8.2.8 using a general time reversible model from the 85% clustering threshold with 46% minimum samples per locus. Support for each node was determined using 100 fast parametric bootstrap replications. Bootstrap values are adjacent to each node. Scale bar represents mean number of base pair substitutions per site. Figure S2: Ultrametric phylogenies created from maximum likelihood phylogeny of Lampsiline mussels (Figure S1; 85%–46%) using TreePL. These trees were trimmed to remove outgroups and retain only a single individual per species. (A) Ancestral state reconstruction of mantle lures using a symmetrical rates model: Grey = presence of a mantle lure (Fig. 2D), Black = no mantle lure. (B) Ancestral state reconstruction of brood lures using a symmetrical rates model: Blue = complex brood lure (Fig. 2F), Red = simple brood lure (Fig. 2E), Yellow = tethered brood lure (Fig. 2H), Green = no brood lure.

10.7717/peerj.12287/supp-2 Supplemental Information 2 R code used in this study The R code that was used for many of the analyses used in this study, including ancestral state reconstructions, SSE models, and interpreting results from BAMM.

10.7717/peerj.12287/supp-3 Supplemental Information 3 Genomic Data as .phy file as output by iPyrad Genomic data output as alignment file generated with an 85% clustering threshold and minimum 46% sample coverage per locus.

## Additional Information and Declarations

### DNA Deposition

The following information was supplied regarding the deposition of DNA sequences: The raw ddRAD reads are available at GenBank: PRJNA704566, SAMN18093783–SAMN18093865. The aligned ddRAD loci used in these analyses is available at Dryad: https://doi.org/10.5061/dryad.c866t1g62.

### Data Availability

The following information was supplied regarding data availability: The ddRAD-seq data that was used in all analyses and the R code used to perform the analyses are available in the Supplemental Information.
