# Supplementary material for: Evolution of diverse host infection mechanisms delineates an adaptive radiation of lampsiline freshwater mussels centered on their larval ecology
Source: PeerJ. 2021 Nov 16;9:e12287. doi: 10.7717/peerj.12287 (PMC8603817; doi:10.7717/peerj.12287)
Supplement: Supplemental Information 1 — Table S1: Summary of samples used in our analyses including; where samples were obtained, host infection mechanism used, primary host fish, and sources cited for determining host use and host infection mechanisms. NCS = North Carolina State University, UF = University of Florida, INHS = Illinois Natural History Survey, and AABC = Alabama Aquatic Biodiversity Center. Table S2 : Summary of the final number of ddRAD-seq loci for each individual at the 85% and 90% clustering similarity threshold and for 25% and 46% minimum samples per loci. Table S3: Displays the AIC, AICc, and log likelihood values for a set of state dependent speciation models performed independently for three different traits: Mantle lure, Brood lure, and broadcast strategy. The four models performed for each trait include a BiSSE model (2 state trait dependent), a HiSSE model (4 state model with two trait states and two hidden states), a 2-state trait independent null model, and a 4 state trait independent null model. Analysis Performed with the topology recovered using 85% clustering threshold and 46% minimum samples per locus. Figure. S1: Maximum likelihood phylogeny of North American lampsiline mussels created with RAxML v8.2.8 using a general time reversible model from the 85% clustering threshold with 46% minimum samples per locus. Support for each node was determined using 100 fast parametric bootstrap replications. Bootstrap values are adjacent to each node. Scale bar represents mean number of base pair substitutions per site. Figure S2: Ultrametric phylogenies created from maximum likelihood phylogeny of Lampsiline mussels (Figure S1; 85%–46%) using TreePL. These trees were trimmed to remove outgroups and retain only a single individual per species. (A) Ancestral state reconstruction of mantle lures using a symmetrical rates model: Grey = presence of a mantle lure (Fig. 2D), Black = no mantle lure. (B) Ancestral state reconstruction of brood lures using a symmetrical rates model: Blue = comp [file peerj-09-12287-s001.pdf]

Supporting Information for

Evolution of diverse host fish infection mechanisms delineates an adaptive radiation of lampsiline freshwater mussels centered on their larval ecology

Trevor L. Hewitt[1*], Amanda E. Haponski[1], and Diarmaid Ó Foighil[1]

[1] University of Michigan
Ecology and Evolutionary Biology
1013 Ruthven Ann Arbor, MI USA 48109
Phone: 517-242-7972, Email: htrevor@umich.edu

PeerJ

Table S1: Summary of samples used in our analyses including; where samples were obtained, host infection mechanism used, primary host fish, and sources cited for determining host use and host infection mechanisms. NCS = North Carolina State University, UF = University of Florida, INHS = Illinois Natural History Survey, and AABC = Alabama Aquatic Biodiversity Center.

Sample name	Species name	Infection Strategy	infection citations	Tissue Source	Museum ID	Host	host citation
Aplic	Amblema plicata	Broadcast (larval threads)	Haag, 2012	T. Hewitt (River Raisin, MI).	306255	Generalist	Haag, 2012
TH_32	Cambarunio taeniata	Mantle lure and simple brood lure	Barnhart et al., 2008; Haag, 2012	NCS	29180	Bass	Gordon et al., 1994
TH131	Cyrtonaias tampicoensis	Broadcast	Zanatta and Murphy, 2007	UF	438173	Gar	Howells, 1997
TH132	Cyrtonaias tampicoensis	Broadcast	Zanatta and Murphy, 2007	UF	438173	Gar	Howells, 1997
TLH59	Epioblasma triquetra	Host Trapping	Barnhart et al., 2008	INHS	36609	Darter/Sculpin	Barnhart et al., 2008; Haag, 2012
Edila2	Eurynia dilatata	Broadcast (larval threads)	Zanatta and Murphy, 2007; Barnhart et al., 2008	T. Hewitt (River Raisin, MI).	306256	Generalist	Ford and Oliver, 2015
Edila1	Eurynia dilatata	Broadcast (larval threads)	Zanatta and Murphy, 2007; Barnhart et al., 2008	T. Hewitt (River Raisin, MI).	306256	Generalist	Ford and Oliver, 2015
TH125	Glebula rotundata	Broadcast	Zanatta and Murphy, 2007	UF	440636	Sunfish	Parker et al., 1984
TH126	Glebula rotundata	Broadcast	Zanatta and Murphy, 2007	UF	440636	Sunfish	Parker et al., 1984
TH_36	Hamiota altilis	Mantle Lure and tethered brood lure	Barnhart et al., 2008	From Paul Johnson (AABC)	306257	Bass/Sunfish	Haag and Warren, 1999
TH_7	Hamiota australus	Tethered brood lure	Barnhart et al., 2008	UF	441239	Bass	Haag, 2012
TH_37	Hamiota perovalis	Tethered brood lure	Barnhart et al., 2008	From Paul Johnson (AABC)	306258	Bass	Haag and Warren, 1997; Haag, 2012
TH152	Hamiota subangulata	Tethered brood lure	Barnhart et al., 2008	UF	438064	Bass	O'Brien and Box, 1999; Haag, 2012
TLH9	Lampsilis bracteata	Mantle lure and simple brood lure	Barnhart et al., 2008	UF	439084	Bass/Sunfish	Haag, 2012
LCFL68	Lampsilis cardium	Mantle lure and simple brood lure	Barnhart et al., 2008	J. Bergner (Flatt River, MI).	NA	Bass	Haag, 2012
LCEE70	Lampsilis cardium	Mantle lure and simple brood lure	Barnhart et al., 2008	T. Hewitt (Eel River, IN).	306259	Bass	Haag, 2012
LCEE71	Lampsilis cardium	Mantle lure and simple brood lure	Barnhart et al., 2008	T. Hewitt (Eel River, IN).	306259	Bass	Haag, 2012
LCFL69	Lampsilis cardium	Mantle lure and simple brood lure	Barnhart et al., 2008	J. Bergner (Flatt River, MI).	NA	Bass	Haag, 2012
LFre2	Lampsilis fasciola	Mantle lure and simple brood lure	Barnhart et al., 2008	T. Hewitt (Huron River, MI).	306260	Bass	Mummert et al., 2003; Haag, 2012
TH_2	Lampsilis fasciola	Mantle lure and simple brood lure	Barnhart et al., 2008	T. Hewitt (River Raisin, MI).	NA	Bass	Mummert et al., 2003; Haag, 2012
TH_77	Lampsilis fasciola	Mantle lure and simple brood lure	Barnhart et al., 2008	T. Hewitt (Huron River, MI).	306260	Bass	Mummert et al., 2003; Haag, 2012
LFHM04	Lampsilis fasciola	Mantle lure and simple brood lure	Barnhart et al., 2008	T. Hewitt (Huron River, MI).	306260	Bass	Mummert et al., 2003; Haag, 2012
LFHM07	Lampsilis fasciola	Mantle lure and simple brood lure	Barnhart et al., 2008	T. Hewitt (Huron River, MI).	306260	Bass	Mummert et al., 2003; Haag, 2012
TLH4	Lampsilis floridensis	Mantle lure and simple brood lure	Barnhart et al., 2008	UF	340525	Bass/Gar	Keller and Ruessler, 1997; Haag, 2012
TH_45	Lampsilis higginsi	Mantle lure and simple brood lure	Barnhart et al., 2008	INHS	49425	Bass/Walleye	Waller and Holland, 1988; Haag, 2012
TH_87	Lampsilis hydiana	Mantle lure and simple brood lure	Barnhart et al., 2008	UF	440994	Bass	Draxler et al., 2006; Haag, 2012
TH_88	Lampsilis hydiana	Mantle lure and simple brood lure	Barnhart et al., 2008	UF	440994	Bass	Draxler et al., 2006; Haag, 2012
TH_5	Lampsilis ornata	Mantle lure and simple brood lure	Barnhart et al., 2008	UF	438031	Bass	Haag and Warren, 2003; Haag, 2012
TH_96	Lampsilis ovata	Mantle lure and simple brood lure	Barnhart et al., 2008; Haag and Warren, 2003	UF	438255	Bass	Williams et al., 2008; Haag, 2012
TH_94	Lampsilis ovata	Mantle lure and simple brood lure	Barnhart et al., 2008; Haag and Warren, 2003	UF	438255	Bass	Williams et al., 2008; Haag, 2012
TH_95	Lampsilis ovata	Mantle lure and simple brood lure	Barnhart et al., 2008; Haag and Warren, 2003	UF	438255	Bass	Williams et al., 2008; Haag, 2012
TH117	Lampsilis radiata	Mantle lure and simple brood lure	Barnhart et al., 2008	UF	439013	Bass and perch	Eads et al., 2015
TH_8	Lampsilis satura	Mantle lure and simple brood lure	Barnhart et al., 2008	UF	441167	Bass	Haag, 2012
TLH51	Lampsilis siliquoidea	Mantle lure and simple brood lure	Barnhart et al., 2008	INHS	25963	Bass	Keller and Ruessler, 1997
TH_23	Lampsilis splendida	Mantle lure and simple brood lure	Barnhart et al., 2008	UF	438354	Bass	Haag, 2012
TLH6	Lampsilis straminea	Mantle lure and simple brood lure	Barnhart et al., 2008	UF	383152	Bass	Keller and Ruessler, 1997; Haag, 2012
TH_38	Lampsilis virescens	Mantle lure and simple brood lure	Barnhart et al., 2008	Paul Johnson (AABC)	306261	Bass	Williams et al., 2008; Haag, 2012
TH_22	Leaunio umbrans	Mantle lure and simple brood lure	Barnhart et al., 2008	UF	438189	Sunfish/Sculpin	Williams et al., 2008
TLH26	Leaunio vanuxemensis	Mantle lure and simple brood lure	Barnhart et al., 2008	UF	438796	Sculpin	Haag, 2012
TH_34	Lemiox rimosus	Mantle lure	Zanatta and Murphy, 2007	NCS	47243	Darter/Sculpin	Haag, 2012
TH_57	Leptodea fragilis	Mantle lure	Sietman et al., 2018	INHS	79830	Drum	Haag, 2012
TH133	Leptodea ochracea	broadcast	Haag et al., 2012	UF	438459	white perch	Wick and Huryn, 2003
TH_91	Ligumia recta	Mantle lure and simple brood lure	Barnhart et al., 2008; Corey et al., 2006	UF	438249	Walleye	Haag, 2012
TH_39	Medionidus acutissimus	Mantle lure	Haag and Warren, 2003	Paul Johnson (AABC)	306262	Darter/Sculpin	Haag and Warren, 1997; Haag and Warren, 2003; Haag, 2012
TH_40	Medionidus conradicus	Mantle lure	Zanatta and Murphy, 2007	UF	438914	Darter/Sculpin	Zale and Neves, 1982; Haag, 2012
TH_41	Medionidus parvulus	Mantle lure	Haag, 2012	Paul Johnson (AABC)	306263	Darter/Sculpin	Haag, 2012
TLH28	Medionidus parvulus	Mantle lure	Haag, 2012	Paul Johnson (AABC)	306263	Darter/Sculpin	Haag, 2012
TH_16	Medionidus penicillatus	Mantle lure	Haag, 2012	Paul Johnson (AABC)	306264	Darter/Sculpin	O'brien and Williams, 2002; Haag, 2012
TLH20	Medionidus simposonianus	Mantle lure	Haag, 2012	Paul Johnson (AABC)	306265	Darter/Sculpin	Haag, 2012

TH_17	Medionidus walkeri	Mantle lure	Haag, 2012	Paul Johnson (AABC)	306266	Darter/Sculpin	Johnson et al., 2016; Haag, 2012
TLH18	Obovaria choctawensis	Mantle lure	Haag, 2012	UF	441237	Darter/Sculpin	Haag, 2012
TH146	Obovaria subrotunda	Mantle lure	Haag, 2012	UF	438391	Darter/Sculpin	Haag, 2012
TH145	Obovaria subrotunda	Mantle lure	Haag, 2012	UF	438391	Darter/Sculpin	Haag, 2012
TH144	Potamilus ohiensis	broadcast	Zanatta and Murphy, 2007; Barnhart et al., 2008	UF	438806	Drum	Haag, 2012
TH143	Potamilus ohiensis	broadcast	Zanatta and Murphy, 2007	UF	438806	Drum	Haag, 2012
TH142	Ptychobranchus fasciolarus	complex brood lure	Barnhart et al., 2008	UF	438254	Darter/Sculpin	Haag, 2012
TH141	Ptychobranchus fasciolarus	complex brood lure	Barnhart et al., 2008	UF	438254	Darter/Sculpin	Haag, 2012
TLH42	Ptychobranchus foremanianus	complex brood lure	Barnhart et al., 2008	Paul Johnson (AABC)	306267	Darter/Sculpin	Haag, 2012
TH153	Ptychobranchus jonesi	complex brood lure	Barnhart et al., 2008	UF	441272	Darter/Sculpin	Haag, 2012
TH_89	Quadrula quadrula	Mantle lure	Barnhart et al., 2008	UF	438787	Catfish	Haag, 2012
TLH21	Sagittunio nasuta	Mantle lure and simple brood lure	Barnhart et al., 2008; Haag, 2012	UF	438285	Sunfish and Perch	Eads et al., 2015
TH147	Sagittunio subrostrata	Mantle lure and simple brood lure	Barnhart et al., 2008	UF	441304	Sunfish	Stern and Felder, 1978
TH148	Sagittunio subrostrata	Mantle lure and simple brood lure	Barnhart et al., 2008	UF	441304	Sunfish	Stern and Felder, 1978
TH_19	Toxolasma corvunculus	Manlte lure	Barnhart et al., 2008	UF	440843	Sunfish	Haag, 2012
TLH44	Toxolasma cylindrellus	Manlte lure	Barnhart et al., 2008; Haag et al. 2012	INHS	49319	Sunfish	Haag, 2012
TH130	Toxolasma lividum	Manlte lure	Roe, 2002; Barnhart et al., 2008	UF	438185	Sunfish	Haag, 2012
TH129	Toxolasma lividum	Manlte lure	Roe, 2002; Barnhart et al., 2008	UF	438185	Sunfish	Haag, 2012
TH128	Toxolasma texasiensis	Manlte lure	Zanatta and Murphy, 2007; Barnhart et al., 2008	UF	438567	Sunfish	Haag, 2012
TH127	Toxolasma texasiensis	Manlte lure	Zanatta and Murphy, 2007; Barnhart et al., 2008	UF	438567	Sunfish	Haag, 2012
TH119	Truncilla macrodon	broadcast	Haag et al., 2012	UF	441301	Drum	Haag, 2012
TH120	Truncilla macrodon	broadcast	Haag et al., 2012	UF	441301	Drum	Haag, 2012
TH122	Truncilla truncata	Manlte lure	Sietman et al., 2018	UF	438976	Drum	Haag, 2012
TH121	Truncilla truncata	Manlte lure	Sietman et al., 2018	UF	438976	Drum	Haag, 2012
TH_63	Venustaconcha ellipsiformis	Mantle lure and simple brood lure	Hove and Anderson, 1997; Barnhart et al., 2008	INHS	87179	Darter/Sculpin	Haag, 2012
TLH25	Venustaconcha trabalis	Mantle lure and simple brood lure	Barnhart et al., 2008	Paul Johnson (AABC)	NA	Darter/Sculpin	Haag, 2012
TH_43	Venustaconcha trabalis	Mantle lure and simple brood lure	Barnhart et al., 2008	UF	438909	Darter/Sculpin	Haag, 2012
TH124	Venustaconcha trabalis	Mantle lure and simple brood lure	Barnhart et al., 2008	UF	438909	Darter/Sculpin	Haag, 2012
TH123	Venustaconcha trabalis	Mantle lure and simple brood lure	Barnhart et al., 2008	UF	438909	Darter/Sculpin	Haag, 2012
TH_11	Villosa amygdala	Mantle lure and simple brood lure	Barnhart et al., 2008	UF	441054	Presumed Bass	
TLH12	Villosa delumbis	Mantle lure and simple brood lure	Barnhart et al., 2008	UF	437984	Bass	Johnson et al., 2002
TH_24	Villosa vibex	Mantle lure and simple brood lure	Barnhart et al., 2008	UF	438545	Sunfish	Haag et al., 1997
TH_13	Villosa vibex	Mantle lure and simple brood lure	Barnhart et al., 2008	UF	438021	Sunfish	Haag et al., 1997
TLH14	Villosa villosa	Mantle lure and simple brood lure	Barnhart et al., 2008	UF	441268	Bass/Sunfish	Keller and Ruessler, 1997

Table S2: Summary of the final number of ddRAD-seq loci for each individual at the 85% and 90% clustering similarity threshold and for 25% and 46% minimum samples per loci.

			85% Similarity		90% Similarity	
Sample name	Species name	Raw Reads	25%	46%	25%	46%
Aplic	Amblema plicata	2900279	301	134	19	8
TH131	Cyrtonaias tampicoensis	858098	208	113	10	3
TH132	Cyrtonaias tampicoensis	1506226	366	170	22	7
TLH59	Epioblasma triquetra	5459944	1678	479	129	18
Edila2	Eurynia dilatata	1726245	203	107	9	4
Edila1	Eurynia dilatata	790501	96	55	5	3
TH125	Glebula rotundata	1070046	303	140	11	4
TH126	Glebula rotundata	2490529	537	216	27	8
TH_36	Hamiota altilus	5387472	1827	497	134	23
TH_7	Hamiota australus	3109960	1494	459	115	19
TH_37	Hamiota perovalis	5270101	1826	513	144	24
TH152	Hamiota subangulata	668819	722	255	62	10
TLH9	Lampsilis bracteata	2568126	1594	476	151	19
LCFL68	Lampsilis cardium	7216326	2545	589	213	21
LCEE70	Lampsilis cardium	6965414	2562	597	215	24
LCEE71	Lampsilis cardium	4974228	2424	590	214	22
LCFL69	Lampsilis cardium	4710408	2470	573	213	23
LFre2	Lampsilis fasciola	1929585	2863	496	163	21
TH_2	Lampsilis fasciola	2188128	3296	560	165	18
TH_77	Lampsilis fasciola	3435913	3816	622	186	24
LFHM04	Lampsilis fasciola	1931502	3403	586	164	18
LFHM07	Lampsilis fasciola	1561315	3216	562	156	17
TLH4	Lampsilis floridensis	3303826	1595	473	107	22
TH_45	Lampsilis higginsi	1009895	512	173	66	12
TH_87	Lampsilis hydiana	2000552	1743	480	175	18
TH_88	Lampsilis hydiana	2183348	1945	519	175	21
TH_5	Lampsilis ornata	4893511	2177	514	161	21
TH_96	Lampsilis ovata	3235994	2320	575	206	23
TH_94	Lampsilis ovata	1807208	1935	526	177	17
TH_95	Lampsilis ovata*	3933699	3232	604	184	23
TH117	Lampsilis radiata	800488	1262	426	120	16
TH_8	Lampsilis satruna	4904722	2328	570	208	23
TLH51	Lampsilis siliquoidea	2111249	1786	498	166	19
TH_23	Lampsilis splendida	1149372	1237	390	129	18
TLH6	Lampsilis straminea	4914716	2123	531	195	22

TH_38	Lampsilis virescens	4169896	2043	527	190	23
TH_34	Lemiox rimosus	1911799	460	200	17	8
TH_57	Leptodea fragilis	3519359	484	204	32	9
TH133	Leptodea ochracea	287978	82	49	4	2
TLH21	Sagittunio nasuta	4608659	1513	452	106	20
TH_91	Ligumia recta	1659317	1382	412	88	15
TH147	Sagittunio subrostrata	1814864	998	352	100	19
TH148	Sagittunio subrostrata	2530822	1184	381	106	20
TH_39	Medionidus acutissimus	1851620	475	205	31	10
TH_40	Medionidus conradicus	7718202	619	226	36	10
TH_41	Medionidus parvulus	9957817	633	233	45	12
TLH28	Medionidus parvulus	6651085	604	228	35	11
TH_16	Medionidus penicillatus	7915534	660	242	43	11
TLH20	Medionidus simposonianus	4362329	583	235	35	12
TH_17	Medionidus walkeri	3139933	559	221	26	8
TLH18	Obovaria choctawensis	1470462	1052	374	98	20
TH146	Obovaria subrotunda	1790649	1238	399	105	18
TH145	Obovaria subrotunda	1672141	1157	383	102	18
TH144	Potamilus ohiensis	2454656	310	148	26	9
TH143	Potamilus ohiensis	2251207	294	142	24	9
TH142	Ptychobranchus fasciolarus	2517640	454	184	28	9
TH141	Ptychobranchus fasciolarus	2576902	494	188	29	9
TLH42	Ptychobranchus foremanianus	14377252	659	224	37	10
TH153	Ptychobranchus jonesi	1455454	355	155	21	7
TH_89	Quadrula quadrula	4999562	148	87	5	3
TH_19	Toxolasma corvunculus	2924381	275	131	15	8
TLH44	Toxolasma cylindrellus	11371070	361	158	27	8
TH130	Toxolasma lividum	817733	43	24	1	0
TH129	Toxolasma lividum	779097	113	65	6	4
TH128	Toxolasma texasiensis	1298761	139	84	6	4
TH127	Toxolasma texasiensis	1454598	204	117	13	5
TH119	Truncilla macrodon	878924	269	152	13	8
TH120	Truncilla macrodon	685468	109	64	10	7
TH122	Truncilla truncata	950716	303	158	18	9
TH121	Truncilla truncata	1613852	421	182	19	8
TH_63	Venustaconcha ellipsiformis	4434860	1702	501	141	20
TLH25	Venustaconcha trabalis	5295838	1825	520	169	21
TH_43	Venustaconcha trabalis	5121294	1829	529	173	23
TH124	Venustaconcha trabalis	2642641	1744	520	166	22
TH123	Venustaconcha trabalis	1660491	1469	495	137	19

TH_11	Villosa amygdala	2021257	1133	377	93	19
TLH12	Villosa delumbis	4433617	1544	447	106	22
TH_32	Cambarunio taeniata	1472633	1004	328	68	16
TH_22	Leaunio umbrans	5607023	1738	492	126	20
TLH26	Leaunio vanuxemensis	1120139	504	188	31	8
TH_24	Villosa vibex	1272879	941	322	74	14
TH_13	Villosa vibex	2427081	1424	454	102	21
TLH14	Villosa villosa	2756754	1340	427	103	22

Table S3: Displays the AIC, AICc, and log likelihood values for a set of state dependent speciation models performed independently for three different traits: Mantle lure, Brood lure, and broadcast strategy. The four models performed for each trait include a BiSSE model (2 state trait dependent), a HiSSE model (4 state model with two trait states and two hidden states), a 2-state trait independent null model, and a 4 state trait independent null model. Analysis Performed with the topology recovered using 85% clustering threshold and 46% minimum samples per locus.

	Mantle Lure			Brood Lure			Broadcast Strategy		
Model Name	AIC	AICc	Log Likelihood	AIC	AICc	Log Likelihood	AIC	AICc	Log Likelihood
**2-state CID**	**544.17**	**545.4**	**-267.0859**	527.25	528.47	-258.8246	518.18	519.43	-254.0909
BiSSE	549.33	550.55	-269.6641	**525.25**	**526.47**	**-257.6246**	**516.72**	**517.98**	**-253.3635**
4-state CID	549.28	553.28	-265.6379	530.2	534.2	-256.0995	517.84	521.93	-249.9179
HiSSE	550.12	554.12	-266.058	532.16	536.16	-257.0816	516.94	521.03	-249.469

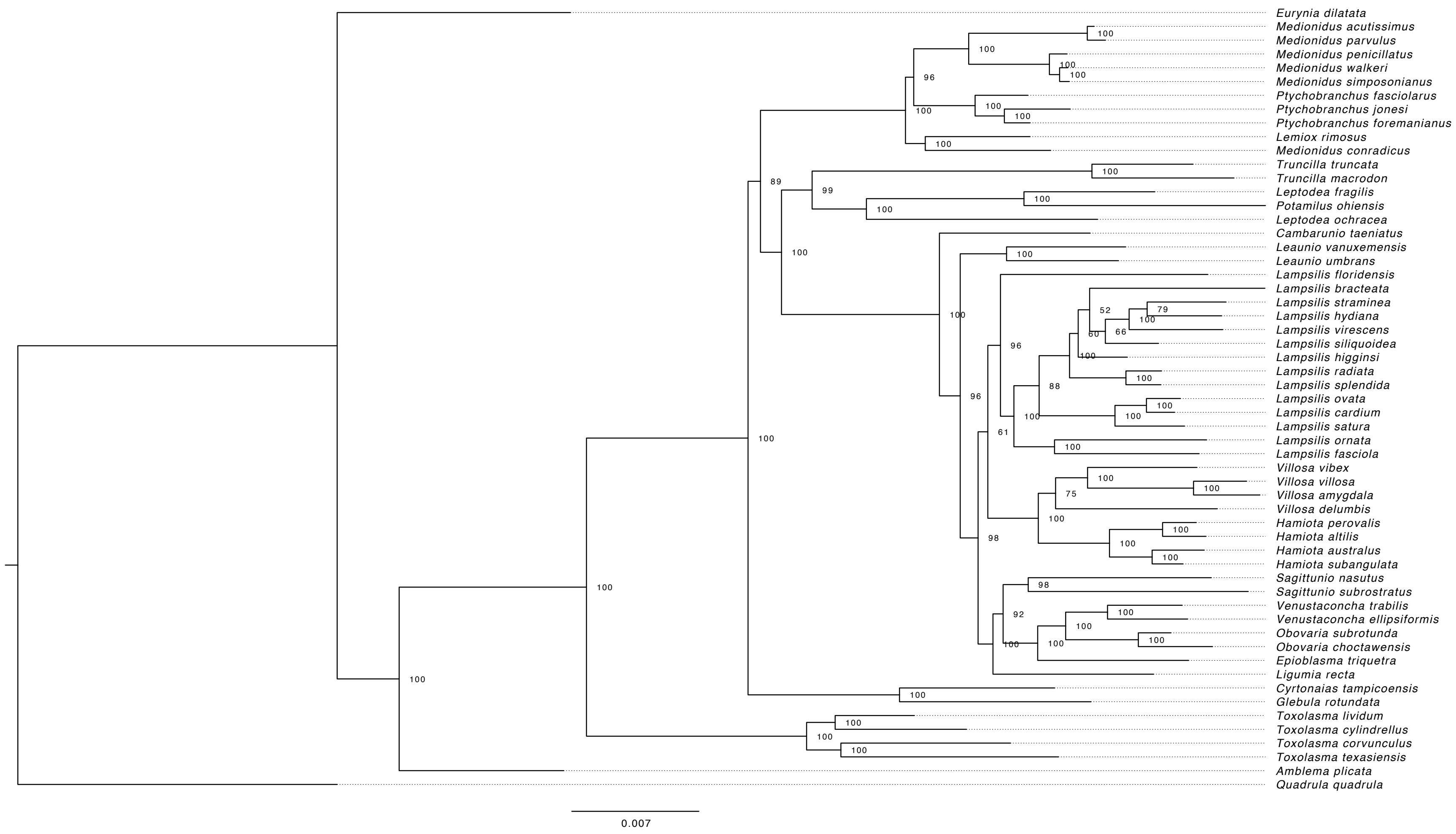

Figure S1: Maximum likelihood phylogeny of North American lampsiline mussels created with RAxML v8.2.8 using a general time reversible model from the 85% clustering threshold with 46% minimum samples per locus dataset. Support for each node was determined using 100 fast parametric bootstrap replications. Bootstrap values are adjacent to each node. Scale bar represents mean number of base pair substitutions per site.

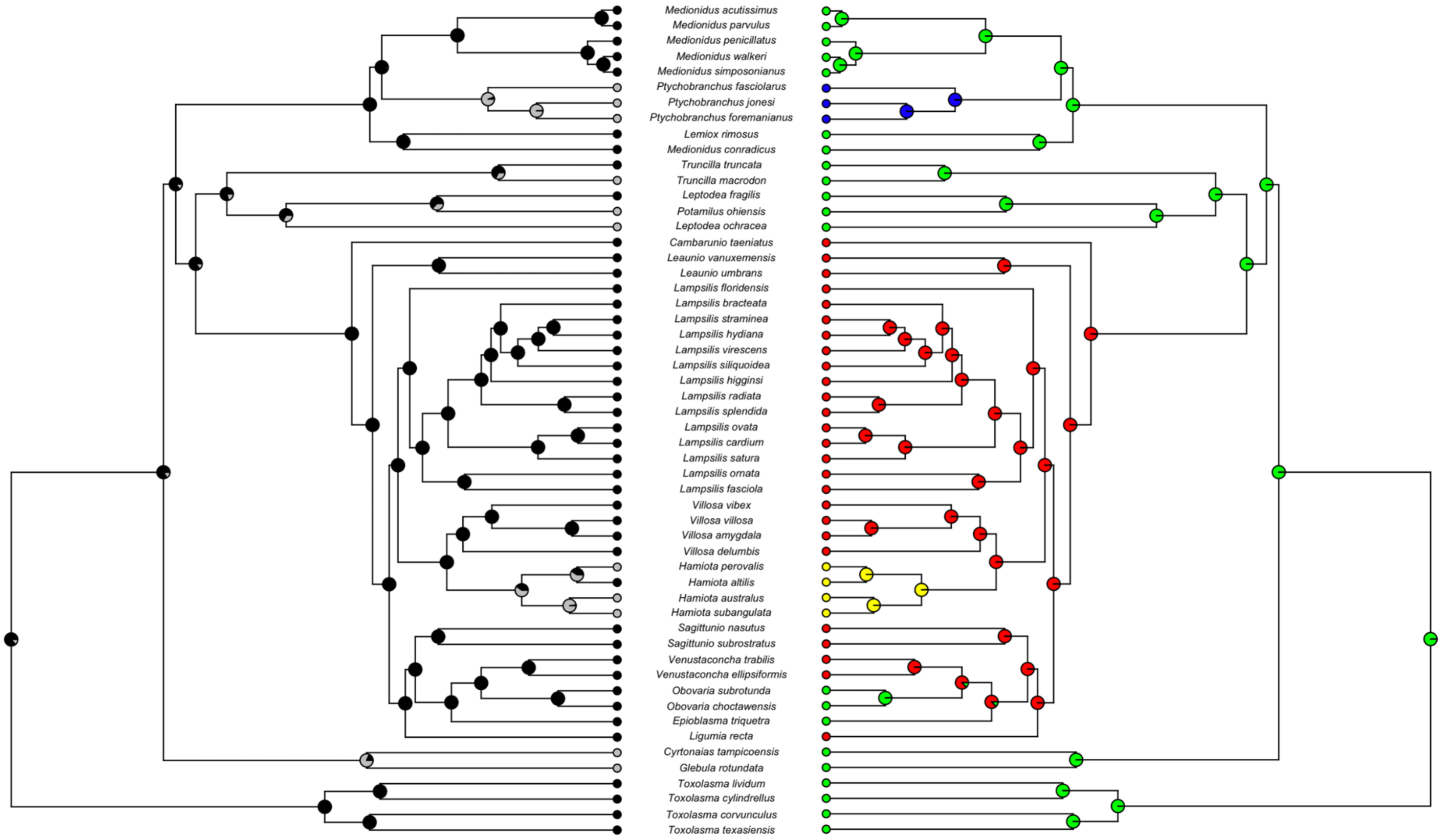

Figure S2: Ultrametric phylogenies created from maximum likelihood phylogeny of Lampsiline mussels (Fig. S1; 85%-46%) using TreePL. These trees were trimmed to remove outgroups and retain only a single individual per species. A) Ancestral state reconstruction of mantle lures using a symmetrical rates model: Grey = presence of a mantle lure (fig. 2d), Black = no mantle lure. B) Ancestral state reconstruction of brood lures using a symmetrical rates model: Blue = complex brood lure (fig. 2f), Red = simple brood lure (fig. 2e), Yellow = tethered brood lure (fig. 2h), Green = no brood lure.

Supplementary data literature cited

Barnhart MC, Haag WR, Roston WN. 2008. Adaptations to host infection and larval parasitism in Unionoida. *Journal of the North American Benthological Society* 27:370–394. DOI: 10.1899/07-093.1.

Corey CA, Dowling R, Strayer DL. 2006. Display Behavior of Ligumia (Bivalvia: Unionidae). *Northeastern Naturalist* 13:319–332.

Cummings KS, Watters GT. 2017. Freshwater Mussel Host Database.

Draxler B, Hove M, Schieffer S, Berg M, Widiker G, Sietman B, Allen D, Hornbach D. 2006. Suitable host fishes for fatmucket (Lampsilis siliquoidea) and pocketbook (Lampsilis cardium) evaluated by high school and university researchers. *Ellipsaria* 8:12.

Eads CB, Price JE, Levine JF. 2015. Fish Hosts of Four Freshwater Mussel Species in the Broad River, South Carolina. *Southeastern Naturalist* 14:85–97. DOI: 10.1656/058.014.0120.

Ford DF, Oliver AM. 2015. The Known and Potential Hosts of Texas Mussels: Implications for Future Research and Conservation Efforts. *Freshwater Mollusk Biology and Conservation* 18:1. DOI: 10.31931/fmbc.v18i1.2015.1-14.

Haag WR. 2012. *North American Freshwater Mussels: Natural History, Ecology, and Conservation*. Cambridge University Press.

Haag WR, Warren, ML. 1997. Host Fishes and Reproductive Biology of 6 Freshwater Mussel Species from the Mobile Basin, USA. *Journal of the North American Benthological Society* 16:576–585. DOI: 10.2307/1468145.

Haag WR, Warren ML. 1999. Mantle displays of freshwater mussels elicit attacks from fish: Mussel and fish interactions. *Freshwater Biology* 42:35–40. DOI: 10.1046/j.1365-2427.1999.00454.x.

Haag WR, Warren, ML. 2003. Host Fishes and Infection Strategies of Freshwater Mussels in Large Mobile Basin Streams, USA. *Journal of the North American Benthological Society* 22:78–91. DOI: 10.2307/1467979.

Hove M, Anderson T. 1997. Mantle-waving behavior and suitable fish hosts of the ellipse. *Triannual Unionid Report* 11:28–29.

Howells RG. 1997. New fish hosts for nine freshwater mussels (Bivalvia: Unionidae) in Texas. 49:255–258.

Johnson N, McLeod J, Holcomb J, Rowe M, Williams J. 2016. Early life history and spatiotemporal changes in distribution of the rediscovered Suwannee moccasinshell Medionidus walkeri (Bivalvia: Unionidae). *Endangered Species Research* 31:163–175. DOI: 10.3354/esr00752.

Johnson JA, Wisniewski JM, Fritts AK, Bringolf RB. 2012. Host Identification and Glochidia Morphology of Freshwater Mussels from the Altamaha River Basin. *Southeastern Naturalist* 11:733–746. DOI: 10.1656/058.011.0411.

Keller AE, Ruessler DS. 1997. Determination or Verification of Host Fish for Nine Species of Unionid Mussels. *American Midland Naturalist* 138:402. DOI: 10.2307/2426831.

Mummert AK, Neves RJ, Newcomb TJ, Cherry DS. 2003. SENSITIVITY OF JUVENILE FRESHWATER MUSSELS (LAMPSILIS FASCIOLA, VILLOSA IRIS) TO TOTAL AND UN-IONIZED AMMONIA. *Environmental Toxicology and Chemistry* 22:2545. DOI: 10.1897/02-341.

O'Brien CA, Box JB. 1999. Reproductive Biology and Juvenile Recruitment of the Shinyrayed Pocketbook, Lampsilis subangulata (Bivalvia: Unionidae) in the Gulf Coastal Plain. *The American Midland Naturalist* 142:129–140. DOI: 10.1674/0003-0031(1999)142[0129:RBAJRO]2.0.CO;2.

O'Brien CA, Williams JD. 2002. Reproductive biology of four freshwater mussels (Bivalvia: Unionidae) endemic to eastern Gulf Coastal Plain drainages of Alabama, Florida, and Georgia. *American Malacological Bulletin* 17:147–158.

Parker RS, Hackney CT, Vidrine MF. 1984. Ecology and Reproductive Strategy of a South Louisiana Freshwater Mussel, Glebula rotundata (Lamarck) (Unionidae:Lampsilini). *Freshwater Invertebrate Biology* 3:53–58. DOI: 10.2307/1467094.

Roe KJ, Harris PM, Mayden RL. 2002. Phylogenetic Relationships of the Genera of North American Sunfishes and Basses (Percoidei: Centrarchidae) as Evidenced by the Mitochondrial Cytochrome *b* Gene. *Copeia* 2002:897–905. DOI: 10.1643/0045-8511(2002)002[0897:PROTGO]2.0.CO;2.

Sietman B, Hove M, Davis M. 2018. Host attraction, brooding phenology, and host specialization on freshwater drum by 4 freshwater mussel species. *Freshwater science* 37:000–000. DOI: 10.1086/696382.

Stern EM, Felder DL. 1978. Identification of Host Fishes for Four Species of Freshwater Mussels (Bivalvia: Unionidae). *American Midland Naturalist* 100:233. DOI: 10.2307/2424795.

Waller DL, Holland Bartels LE. 1988. Fish hosts for glochidia of the endangered freshwater mussel Lampsilis higginsi Lea (Bivalvia: Unionidae). *Malacological Review* 21:119–122.

Wick P, Huryn A. 2002. Biology and natural history of Lampsilis cariosa and Leptodea ochracea (Unionidae) in Maine. *Bulletin of the North American Benthological Society* 19:175–176.

Williams JD, Bogan AE, Garner JT. 2008. *Freshwater mussels of Alabama and the Mobile basin in Georgia, Mississippi, and Tennessee*. University of Alabama Press.

Zale AV, Neves RJ. 1982. Fish hosts of four species of lampsiline mussels (Mollusca: Unionidae) in Big Moccasin Creek, Virginia. *Canadian Journal of Zoology* 60:2535–2542.

Zanatta DT, Murphy RW. 2006. Evolution of active host-attraction strategies in the freshwater mussel tribe Lampsilini (Bivalvia: Unionidae). *Molecular Phylogenetics and Evolution* 41:195–208. DOI: 10.1016/j.ympev.2006.05.030.
